# Expression and Gene Regulation Network of Adenosine Receptor A2B in Lung Adenocarcinoma: A Potential Diagnostic and Prognostic Biomarker

**DOI:** 10.3389/fmolb.2021.663011

**Published:** 2021-07-19

**Authors:** Yutong Sui, Jiayin Liu, Jing Zhang, Zena Zheng, Ziwei Wang, Zhenghu Jia, Ziyu Meng

**Affiliations:** ^1^ Shenzhen Hospital, Southern Medical University, Shenzhen, China; ^2^ Department of Radiation Oncology, Harbin Medical University Cancer Hospital, Harbin, China; ^3^ The First Affiliated Hospital, Biomedical Translational Research Institute and Guangdong Province Key Laboratory of Molecular Immunology and Antibody Engineering, Jinan University, Guangzhou, China; ^4^ NHC Key Laboratory of Hormones and Development, Tianjin Key Laboratory of Metabolic Diseases, Chu Hsien-I Memorial Hospital and Tianjin Institute of Endocrinology, Tianjin Medical University, Tianjin, China

**Keywords:** *ADORA2B*, lung adenocarcinoma, mRNA expression, functional network analysis, verification experiment

## Abstract

*Adenosine* *receptor A2B* (*ADORA2B*) encodes a protein belonging to the G protein–coupled receptor superfamily. Abnormal expression of *ADORA2B* may play a pathophysiological role in some human cancers. We investigated whether ADORA2B is a potential diagnostic and prognostic biomarker for lung adenocarcinoma (LUAD). The expression, various mutations, copy number variations, mRNA expression levels, and related network signaling pathways of *ADORA2B* were analyzed using bioinformatics-related websites, including Oncomine, UALCAN, cBioPortal, GeneMANIA, LinkedOmics, KM Plotter, and TIMER. We found that *ADORA2B* was overexpressed and amplified in LUAD, and a high *ADORA2B* expression predicted a poor prognosis for LUAD patients. Pathway analyses of *ADORA2B* in LUAD revealed *ADORA2B*-correlated signaling pathways, and the expression level of *ADORA2B* was associated with immune cell infiltration. Furthermore, *ADORA2B* mRNA and protein levels were significantly higher in human LUAD cell lines (A549 cells and NCl-H1299 cells) than in normal human bronchial epithelial (HBE) cells, and the transcript levels of genes positively or negatively correlated with *ADORA2B* were consistent and statistically significant. siRNA transfection experiments and functional experiments further confirmed these results. *In vitro* results were also consistent with those of bioinformatics analysis. Our findings provide a foundation for studying the role of *ADORA2B* in tumorigenesis and support the development of new drug targets for LUAD.

## Introduction

Lung adenocarcinoma (LUAD) is a common pathological form of lung cancer and is associated with high mortality, leading to more than one million deaths worldwide every year (TCGA, 2018). LUAD is characterized by the presence of heterogeneous tumors that originate in the small airway and spread to the surrounding lung tissue (Yu et al., 2010; Russell et al., 2011). Multiple organ metastasis is the leading cause of death in LUAD patients. Within a few months after diagnosis, metastasis to different organs can occur rapidly. Traditional modalities, such as radiotherapy and chemotherapy, are ineffective for LUAD (Zagryazhskaya et al., 2015), and targeted gene therapy holds potential for the clinical management of LUAD. Epidermal growth factor receptor (*EGFR*) mutation and anaplastic lymphoma kinase (*ALK*) fusion are the common gene abnormalities reported in LUAD. Personalized therapy targeting these genes has become a well-designed standard treatment, with erlotinib and gefitinib being the main drugs for LUAD treatment (Yu et al., 2013). Furthermore, mutations in Kirsten rat sarcoma viral oncogene (*KRAS*), human epidermal growth factor receptor 2 (*HER2*), and *V-raf* murine sarcoma viral oncogene homolog B1 (*BRAF*) as well as other fusions of driver genes, such as rearranged during transfection (*RET*) and ROS proto-oncogene 1 (*ROS1*), have been reported in LUAD (Roman et al., 2018; Pillai et al., 2017; Mazieres et al., 2020; Drilon, et al., 2020; Shaw, et al., 2017). These driver genes vary depending on race, sex, and smoking status. The pathogenesis of LUAD is complex and mainly involves cell cycle regulation and signal transduction, reflected in altered gene function at different developmental stages of the disease. New drug targets for LUAD should be identified by screening gene networks associated with tumor formation and progression.

Most of the extracellular adenosine is released from adenine nucleotides in response to various stimuli, including mechanical stress, osmotic stimulation, inflammation, and tissue damage (Borea et al., 2016; Covarrubias et al., 2016; Hamidzadeh and Mosser, 2016). Extracellular adenosine binds to four adenosine receptor subtypes, namely, A1, A2A, A2B, and A3, each of which exhibits unique pharmacological and coupling effects and tissue distribution characteristics. Adenosine receptor A2B (*ADORA2B*) encodes an adenosine receptor belonging to the G protein–coupled receptor superfamily and is highly expressed in various carcinomas, which leads to the promotion of carcinoma cell proliferation and metastasis (Kasama et al., 2015; Vecchio et al., 2016). *ADORA2B* also regulates the tumor microenvironment and is involved in peripheral vascular growth, inflammatory cell infiltration, fibroblast proliferation, and extracellular matrix accumulation (Csoka et al., 2012). Therefore, *ADORA2B* regulates tumor progression and metastasis and could serve as a useful target for cancer therapy or combination therapy.

Recent studies have suggested that ADORA2B is an indispensable target in the regulation of both acute and chronic pulmonary diseases, such as pulmonary hypertension, chronic obstructive pulmonary disease, and pulmonary fibrosis (Karmouty-Quintana et al., 2013; Philip et al., 2017; Mertens et al., 2018; Tian et al., 2021). LUAD is one of the most lethal pulmonary diseases, with the highest mortality rate; however, the role of *ADORA2B* in LUAD is still unknown. In this study, we analyzed the expression and mutations of *ADORA2B* in patients with LUAD using data obtained from various public databases. We also analyzed the genomic variations, survival rates, and functional networks associated with *ADORA2B* in LUAD. Additionally, human LUAD and normal bronchial epithelial cell lines were used to confirm the role of *ADORA2B* in LUAD. siRNA transfection experiments and functional experiments further confirmed the obtained results. Therefore, we used bioinformatics analysis in combination with *in vitro* experiments to comprehensively investigate the role of *ADORA2B* in LUAD.

## Materials and Methods

### Oncomine Analysis

The mRNA expression of *ADORA2B* in LUAD was analyzed using the Oncomine database (www.oncomine.org). LUAD-based databases established from the Okayama Lung, Landi Lung, Selamat Lung, Stearman Lung, Beer Lung, Su Lung, Bhattacharjee Lung, and Garber Lung datasets were used for this analysis (Bhattacharjee et al., 2001; Garber et al., 2001; Beer et al., 2002; Stearman et al., 2005; Su et al., 2007; Landi et al., 2008; Okayama et al., 2012; Selamat et al., 2012). The difference in the expression of *ADORA2B* between LUAD tissues and para-carcinoma tissues was considered statistically significant at *p* < 0.01.

### UALCAN Analysis

Multiple clinical and pathological features related to the mRNA expression of *ADORA2B* in LUAD were analyzed by UALCAN (http://ualcan.path.uab.edu). *ADORA2B* expression was analyzed by TCGA Level 3 RNA sequencing in UALCAN, and the clinical data were analyzed to determine *ADORA2B* expression levels in different tumor subgroups (Chandrashekar et al., 2017).

### cBioPortal Analysis

cBioPortal (http://cbioportal.org) was used to analyze *ADORA2B* alterations observed in The Cancer Genome Atlas (TCGA) LUAD samples. By analyzing various types of mutations, copy number variations, and mRNA expression levels, the tab OncoPrint displayed an overview of the genetic changes in each sample as gene mutations and heat maps of *ADORA2B* expression. The tab plots displayed an overview of the data type of clinical attributes and mutations, including clinical attributes and group mutations by mutation type.

### GeneMANIA Analysis

GeneMANIA (http://genemania.org/) is a web interface that uses large sets of functional association data to identify single genes related to a set of input genes, generating hypotheses on gene function, analyzing gene lists, and prioritizing genes for functional assays. GeneMANIA was used to construct the *ADORA2B* software for the analysis (Warde-Farley et al., 2010).

### LinkedOmics Analysis

The LinkFinder module of LinkedOmics (http://www.linkedomics.org/login.php) was used to identify differentially expressed genes related to *ADORA2B* (*n* = 515) in the TCGA LUAD section. The search and target datasets were obtained by RNA-seq, and the results were analyzed with the Pearson correlation coefficient. The data from the LinkFinder results were labeled and sorted before enrichment analysis was performed for Gene Ontology, KEGG, PANTHER, and Reactome pathway analyses.

### Survival Analysis

We used the Kaplan–Meier (KM) Plotter (http://kmplot.com), an online database that contains gene expression data and survival information of 865 LUAD patients, to analyze the prognostic value of ADORA2B in LUAD. The patient samples were separated into two groups by median expression (high expression and low expression) to analyze the overall survival (OS) with hazard ratios (HRs) with 95% confidence intervals and log-rank *p*-values.

### Immune Infiltration Analysis

Tumor Immune Estimation Resource (TIMER, https://cistrome.shinyapps.io/timer/) is a web resource for systematic analysis of the infiltration levels of different subsets of immune cells in different types of cancers. We analyzed the expression of *ADORA2B* in LUAD in relation to tumor purity and the abundance of immune infiltrating cells including B cells, CD8^+^ T cells, CD4^+^ T cells, macrophages, neutrophils, and dendritic cells. We also analyzed the relationship between the gene copy number variation and the abundance of immune infiltrating cells.

### Cell Culture

A549 cells (human LUAD, ATCC®CCL-185™), NCl-H1299 cells (human LUAD, ATCC®CRL-5803™), and HBE cells (human bronchial epithelial cells, ATCC®CRL-2741™) were obtained from ATCC (Manassas, VA, United States). A549 cells were cultured in Dulbecco’s modified Eagle’s medium (DMEM, Gibco, Grand Island, NY, United States) containing 10% fetal bovine serum (FBS, Gibco), whereas the culture medium for HBE cells contained 20% FBS. NCl-H1299 cells were cultured in RPMI-1640 medium (Gibco, Grand Island, NY, United States) containing 10% FBS. All the cells were incubated in a humidified incubator at 5% CO_2_ and 37°C. The cells that passaged less than five with >70% confluency were used for experiments.

### RNA Extraction and Quantitative Real-time PCR (qRT-PCR)

RNA from A549, NCl-H1299, and HBE cells was isolated using TRIzol Reagent (Invitrogen, Carlsbad, CA, United States) according to the manufacturer's instructions. cDNA synthesis was performed using a cDNA First Strand Synthesis Kit (ABclonal, Wuhan, China), and mRNA expression was quantified by qRT-PCR using the SYBR Green Fast qPCR Mix (ABclonal). Relative mRNA expression levels were normalized to those of β-actin and measured by the comparative Ct (2^−ΔΔCt^) method (*n* = 6 samples/group). Primer sequences used in this study are listed in Supplementary Table S1.

### siRNA Transfection

*ADORA2B* siRNAs and negative control siRNA (50 nM) were obtained from GenePharma (Shanghai, China). The siRNAs were transfected with Lipofectamine 2000 (Invitrogen) for 4 h, following the manufacturer’s protocol. *ADORA2B* knockdown efficiencies were verified by RT-PCR. The siRNA sequences are listed in Supplementary Table S1.

### Western Blotting

A549, NCl-H1299, and HBE cells were cultured as previously described (*Cell Culture*). Total protein was extracted using RIPA buffer (Solarbio, Beijing, China). Protein concentrations were measured using a BCA protein assay kit (Solarbio). Protein extracts (50 μg/well) were separated by 10% sodium dodecyl sulfate polyacrylamide gel electrophoresis and transferred onto polyvinylidene fluoride membranes. The membranes were blocked with 5% skim milk for 2 h and incubated with anti-β-actin (Bioss, bs-0061R, Beijing, China), anti-*ADORA2B* (Bioss, bs-5900R), anti-cyclin D1 (ABclonal, A2708), anti-PCNA (Proteintech, 10205-2-AP, Wuhan, China), anti-N-cadherin (ABclonal, A0433), and anti-vimentin (ABclonal, A2584) antibodies overnight. The next day, the membranes were incubated with a HRP-conjugated secondary antibody and goat anti-rabbit IgG antibody (ABclonal) for 1 h. A western blot detection ECL kit (Advansta, Menlo Park, United States) was used to detect protein bands. Fold-changes in *ADORA2B* protein expression were normalized to those of β-actin.

### Cell Proliferation Assay

Cell proliferation was measured using cell counting kit-8 (CCK-8, Beyotime, Beijing, China). A549 cells (3000 cells/well) were plated in a 96-well plate and transfected with *ADORA2B* siRNA-2 and negative control siRNA as previously described (*siRNA Transfection*). The cell proliferation rate was detected with CCK-8 at 24, 48, and 72 h. The absorbance at 450 nm was measured using a microplate reader.

### Cell Migration Assay

The cells were transfected with *ADORA2B* siRNA-2 and negative control siRNA using Lipofectamine 2000 as previously described. Transfected cells were grown to 100% confluence, and then wounds were created using pipette tips. Serum-free medium was used instead of complete medium to eliminate the effects of cell proliferation. Images were captured at 0 and 48 h using a microscope. The cell migration rate was calculated by ImageJ software.

### Statistical Analysis

Data are presented as the mean ± standard error of the replicates. Comparisons between two groups were performed using Student's *t*-test. GraphPad Prism 8.0 software (GraphPad, Inc., La Jolla, CA, United States) was used for statistical analysis. Results with *p* < 0.05 were considered statistically significant (*p < 0.05; **p < 0.01; ***p < 0.001).

## Results

### Expression of *ADORA2B* in LUAD

**FIGURE 1:**
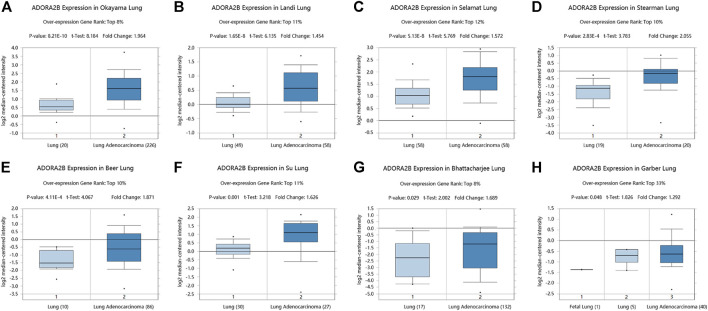
*ADORA2B* transcription in LUAD. *ADORA2B* mRNA copy numbers were significantly higher in LUAD in **(A)** Okayama Lung, **(B)** Landi Lung, **(C)** Selamat Lung, **(D)** Stearman Lung, **(E)** Beer Lung, **(F)** Su Lung, **(G)** Bhattacharjee Lung, and **(H)** Garber Lung datasets than in para-carcinoma tissues.

We determined *ADORA2B* transcript levels in LUAD patients using data obtained from TCGA and Gene Expression Omnibus (GEO). Data from the Oncomine database showed that the expression of *ADORA2B* mRNA was significantly upregulated in LUAD tissues when compared with that in para-carcinoma tissues (*p* < 0.01). *ADORA2B* ranked in the top 33% of genes with high mRNA expression, with fold-difference values <2 (Figures 1A–H).

**FIGURE 2:**
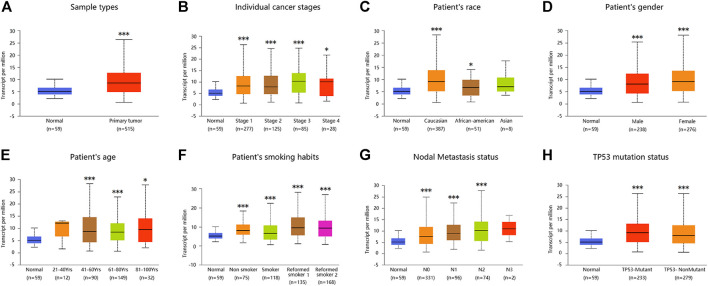
*ADORA2B* expression level in subgroups of patients with LUAD. Relative expression of ADORA2B in normal and different classifications of LUAD tissues. **(A)** Sample types, **(B)** individual cancer stages, **(C)** patient’s race, **(D)** patient’s gender, **(E)** patient’s age, **(F)** patient’s smoking habits, **(G)** nodal metastasis status, and **(H)** TP53 mutation status. *p < 0.05 and ***p < 0.001 are statistically significant.

Similarly, analysis of multiple clinical and pathological features of LUAD patients from the TCGA database revealed elevated *ADORA2B* mRNA expression. Moreover, *ADORA2B* mRNA expression was significantly higher in LUAD tissues than in para-carcinoma tissues in subgroup analyses based on sample type, individual cancer stage, ethnicity, gender, age, smoking habits, nodal metastasis status, and TP53 mutation status (Figures 2A–H).

### Frequency and Type of ADORA2B Alterations in LUAD

**FIGURE 3:**
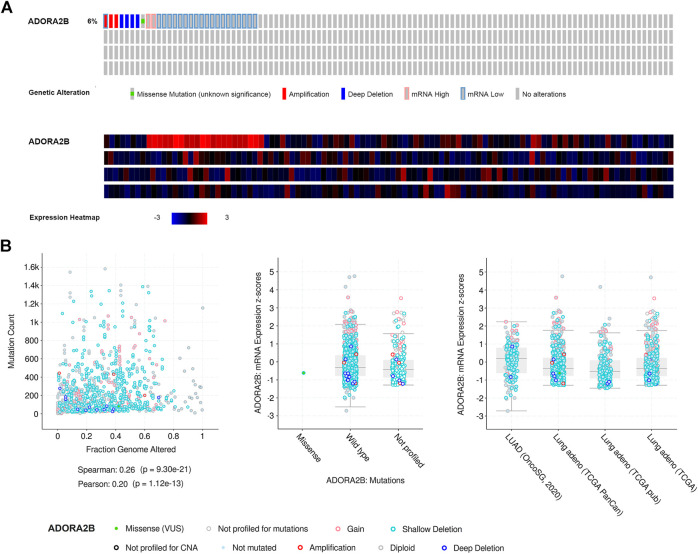
Frequency and type of *ADORA2B* alterations in LUAD. **(A)** OncoPrint of *ADORA2B* alterations in LUAD (cBioPortal) and **(B)** plots of *ADORA2B* alterations in LUAD with clinical attributes and mutations (cBioPortal).

Next, we used the cBioPortal database to evaluate the types and frequencies of *ADORA2B* alterations in LUAD tissues based on sequencing data from patients with LUAD obtained from TCGA’s Pan-Cancer Atlas database. *ADORA2B* was altered in 30 out of 503 (6%) patients with LUAD (Figure 3A). These genetic alterations included missense mutations (1, 0.2%), amplification (3, 0.6%), deep deletion (4, 0.8%), mRNA high (2, 0.4%), and mRNA low (20, 4%). The patient order in OncoPrint is shown in Supplementary Table S2. Analysis included the type of clinical attribute and mutation, fraction of genome altered, and mutation type, including missense (VUS), not mutated, not profiled for mutations, amplification, gain, diploid, shallow deletion, deep deletion, and not profiled for CNA (Figure 3B). mRNA low, shallow deletion, and diploid were the most common types of *ADORA2B* genetic alterations found in LUAD.

### Expression of *ADORA2B* Is Associated With the Survival of LUAD Patients

**FIGURE 4:**
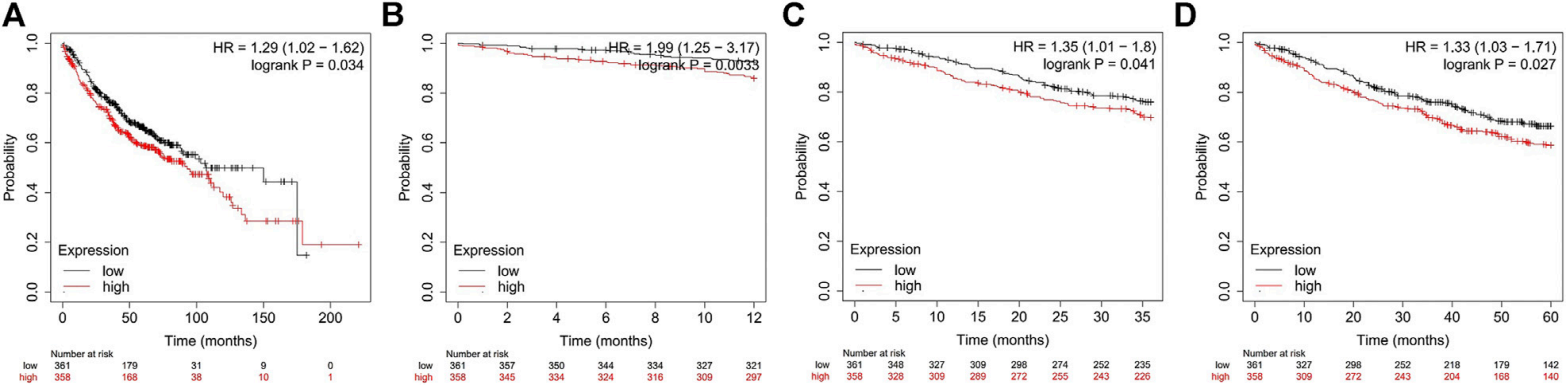
The expression of *ADORA2B* is associated with LUAD patient survival. **(A)** Mean OS analysis of LUAD patients with high and low *ADORA2B* expression OS analysis of LUAD patients with high and low ADORA2B expressions for **(B)** 12 months (1 year), **(C)** 36 months (3 years), and **(D)** 60 months (5 years).

Previous studies have demonstrated that *ADORA2B* participates in the proliferation and metastasis of carcinomas (Kasama et al., 2015; Vecchio et al., 2016). Thus, we further examined the prognostic value of ADORA2B in LUAD patients. LUAD patients with a higher expression of *ADORA2B* exhibited poor OS according to the KM Plotter database. Survival analysis showed that the mean OS time in the low *ADORA2B* expression group was longer than that in the high *ADORA2B* expression group (*p* = 0.034) (Figure 4A). The mean OS times for 12 (1 year), 36 (3 years), and 60 months (5 years) in the low *ADORA2B* expression group were significantly longer than those in the high expression group (Figures 4B–D). These results indicated that *ADORA2B* was significantly associated with the prognosis of LUAD patients and that a high *ADORA2B* expression predicted a poor prognosis in LUAD patients.

### Interaction Network of *ADORA2B*

**FIGURE 5:**
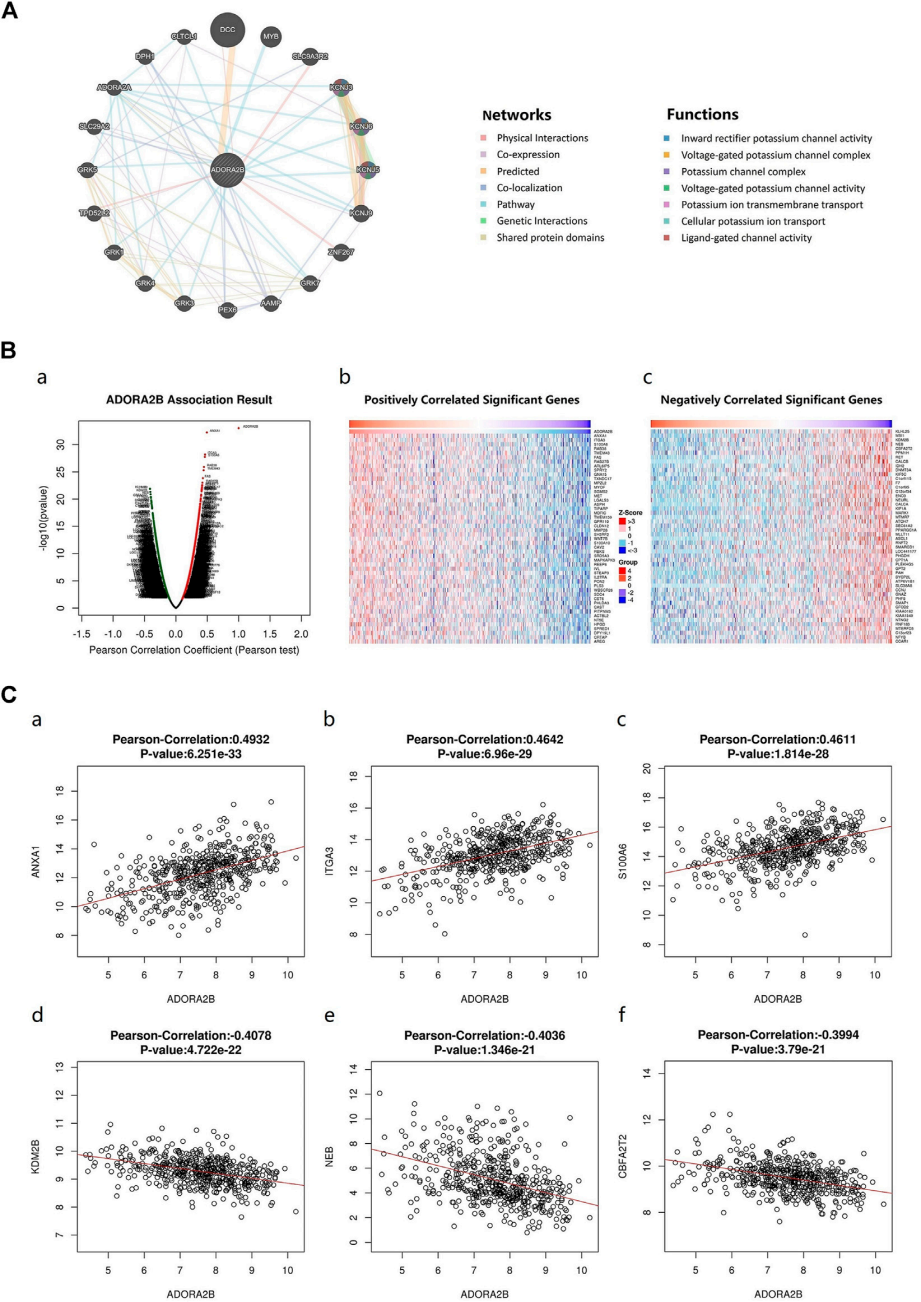
Genes associated with *ADORA2B* expression. **(A)** Biological interaction network of *ADORA2B*. **(B)** Differentially expressed genes correlated with *ADORA2B* (LinkedOmics). (a) Correlations between *ADORA2B* and differentially expressed genes in LUAD were analyzed with Pearson’s correlation coefficient. (b) Heat map of genes positively correlated with *ADORA2B* in LUAD. (c) Heat map of genes negatively correlated with *ADORA2B* in LUAD. **(C)** Gene expression correlation analysis of *ADORA2B* and *ADORA2B*-correlated genes (LinkedOmics). Pearson correlation of *ADORA2B* expression with that of (a) *ANXA1*, (b) *ITGA3*, (c) *S100A6*, (d) *KDM2B*, (e) *NEB*, and (f) *CBFA2T2* (*n* = 515).

An *ADORA2B* correlation network was constructed to determine the potential mutual effects between *ADORA2B* and cancer-related targets. The results indicated that *ADORA2B* could interact with netrin 1 receptor (*DCC*), solute carrier family 9 member A3 regulator 2 (*SLC9A3R2*), zinc finger protein 267 (*ZNF267*), and tumor protein D52-like 2 (*TPD52L2*); *ADORA2B* was co-expressed with potassium inwardly rectifying channel subfamily J member 3 (*KCNJ3*), angio-associated migratory cell protein (*AAMP*), *TPD52L2*, and solute carrier family 29 member 2 (*SLC29A2*); and *ADORA2B* was co-located with *AAMP*, peroxisomal biogenesis factor 6 (*PEX6*), *SLC29A2*, *ADORA2A*, and diphthamide biosynthesis 1 (*DPH1*). *ADORA2B* was identified to interact with *DCC* and shared protein domains with its isoform *ADORA2A* (Figure 5A).

The LinkedOmics database was used to identify RNA-seq genes co-expressed with *ADORA2B* in LUAD. A volcano plot indicated that the expression of 5,246 genes was negatively correlated with *ADORA2B* expression, whereas the expression of 3,447 genes was positively correlated with *ADORA2B* expression. We confirmed the top 50 genes that negatively or positively interacted with *ADORA2B* (Figure 5B and Supplementary Table S3). The results revealed that multiple differentially expressed genes were correlated with *ADORA2B* expression.

Pearson's correlation coefficient analysis revealed a positive correlation between *ADORA2B* expression levels and those of annexin A1 (*ANXA1*), integrin subunit alpha 3 (*ITGA3*), and S100 calcium binding protein A6 (*S100A6*) and a negative correlation between the expression of *ADORA2B* and that of lysine demethylase 2B (*KDM2B*), nebulin (*NEB*), and *CBFA2/RUNX1* partner transcriptional co-repressor 2 (*CBFA2T2*) (Figure 5C).

### Enrichment Analysis of *ADORA2B* Functional Networks in LUAD

**FIGURE 6:**
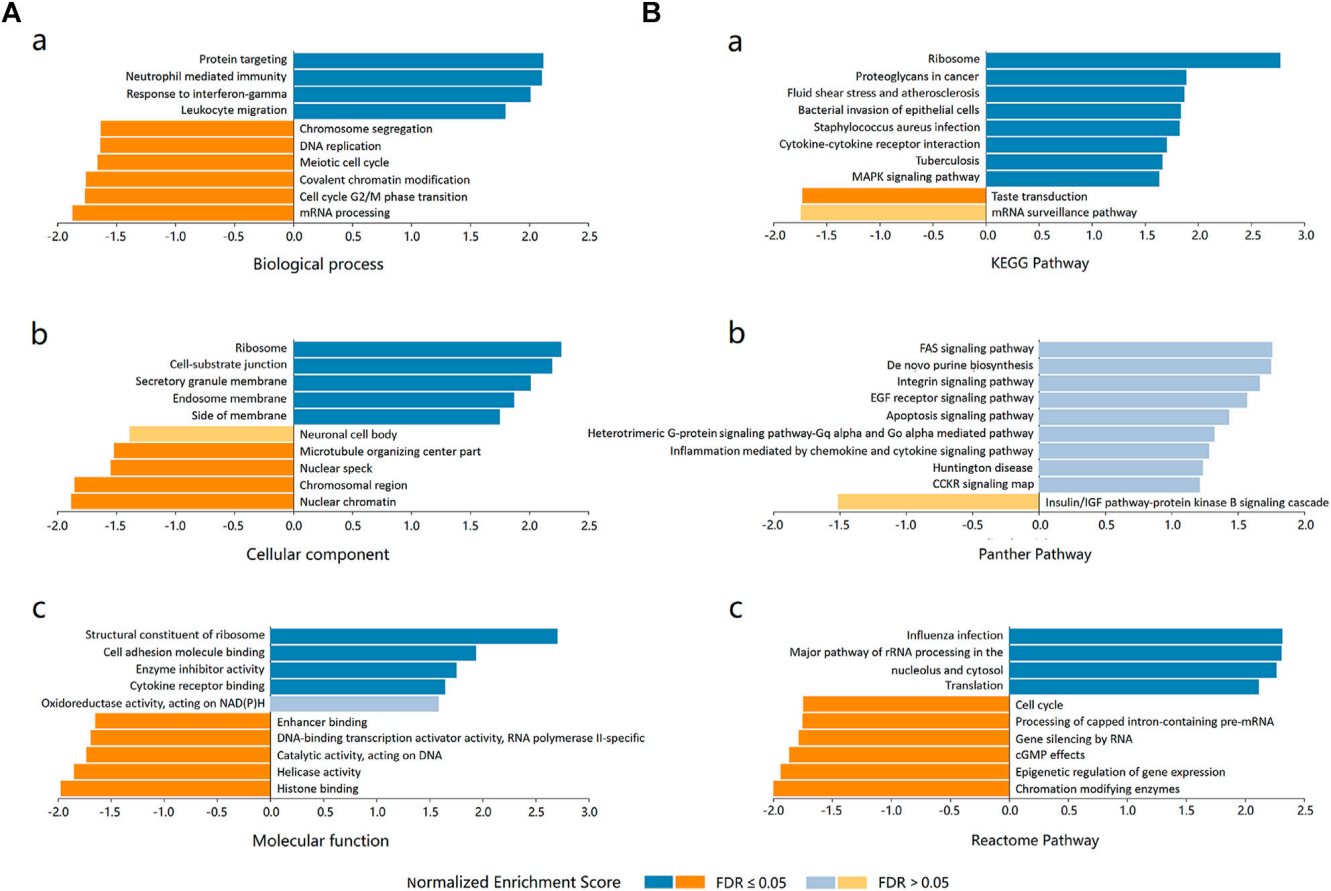
Enrichment analysis of ADORA2B functional networks in LUAD. **(A)** Enriched Gene Ontology annotations of *ADORA2B*-correlated genes in LUAD (LinkedOmics). (a) Biological process. (b) Cellular component. (c) Molecular function. **(B)** Enrichment pathway analysis of *ADORA2B*-correlated genes in LUAD (LinkedOmics). (a) KEGG pathway. (b) PANTHER pathway. (c) Reactome pathway.

Three independent ontologies (biological process, cellular component, and molecular function) were analyzed by gene set enrichment analysis (GSEA). The results indicated that *ADORA2B*-associated differentially expressed genes were involved in several biological processes (protein targeting, neutrophil mediated immunity, response to IFN-γ, etc.), cellular components (ribosome, cell-substrate junction, secretory granule membrane, etc.), and molecular functions (structural constituent of ribosome, cell adhesion molecule binding, enzyme inhibitor activity, etc.) (Figure 6A).

The differentially expressed genes associated with *ADORA2B* were then evaluated for potential functional pathways using KEGG (ribosome, proteoglycans in cancer, fluid shear stress, and atherosclerosis, etc.), PANTHER (FAS, EGF receptor, apoptosis, etc.), and Reactome (influenza infection, major pathway of rRNA processing in the nucleolus and cytosol, cell cycle, etc.) pathway analyses (Figure 6B).

### Relationship Between *ADORA2B* Expression and Infiltrating Immune Cells in LUAD

**FIGURE 7:**
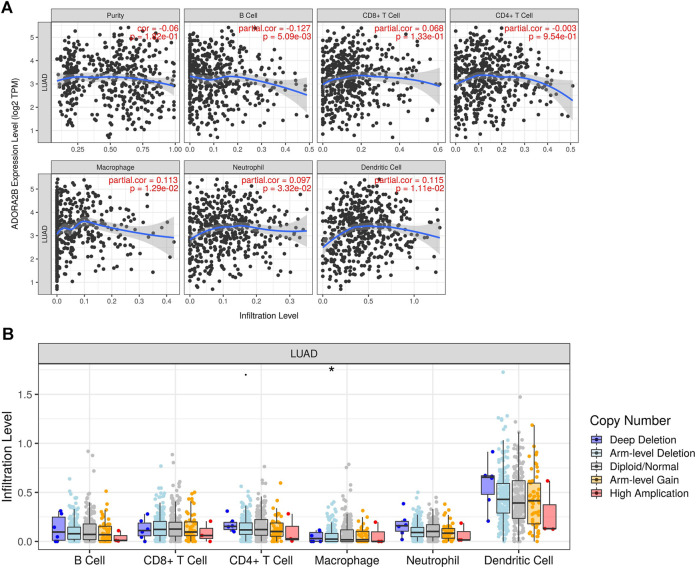
Correlation of *ADORA2B* expression with immune cell infiltration in LUAD. **(A)** Correlation of *ADORA2B* with tumor purity and infiltration of different subsets of immune cells. **(B)** Effect of copy number variation of *ADORA2B* on the distribution of various immune cells. *p < 0.05 is statistically significant.

The tumor microenvironment is a very complex system composed of a variety of cells, enzymes, cytokines, and metabolites and is characterized by low oxygen, low pH, and high pressure. An important immunosuppressive mechanism is mediated by the CD73–adenosine receptor metabolic signaling pathway (Chen et al., 2020). To comprehensively investigate the role of *ADORA2B* in LUAD, we selected the TIMER database to analyze the association of *ADORA2B* expression levels with subsets of infiltrating immune cells. The expression of *ADORA2B* was significantly correlated with the infiltration of B cells, macrophages, neutrophils, and dendritic cells (Figure 7A). In addition, the copy number variation (CNV) of *ADORA2B* was significantly correlated with the infiltration levels of macrophages (Figure 7B).

### Expression Levels of *ADORA2B* and Correlated Genes in Human LUAD Cells

**FIGURE 8:**
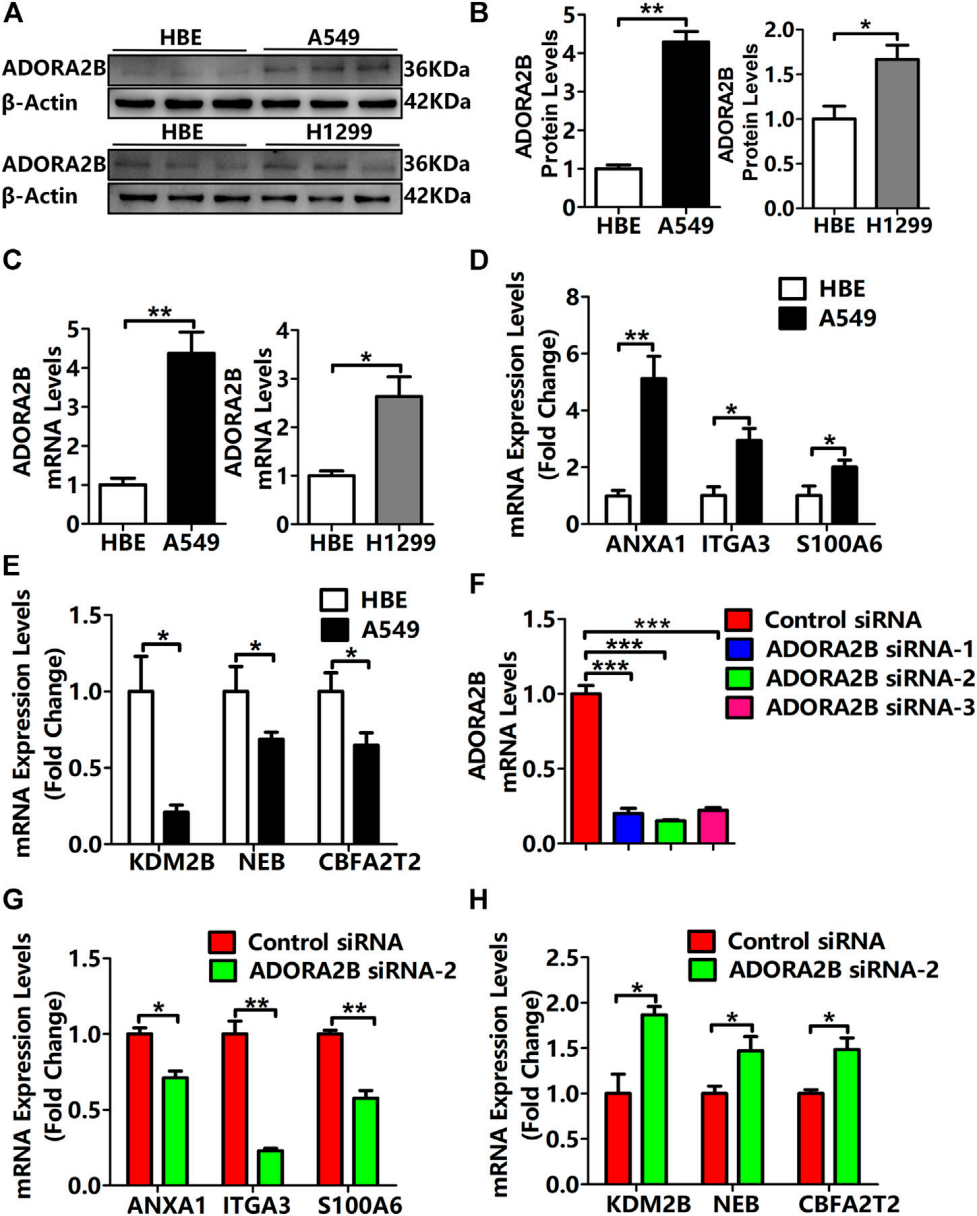
Expression levels of *ADORA2B* and correlated genes in human LUAD cells. **(A)** Relative protein expression of ADORA2B was determined by western blotting. **(B)** Protein quantitative statistics. **(C)** Relative mRNA expression of *ADORA2B* was determined by qRT-PCR. mRNA expression levels of genes **(D)** positively and **(E)** negatively correlated with *ADORA2B*. **(F)** mRNA expression levels of *ADORA2B* after siRNA transfection. mRNA expression levels of genes **(G)** positively and **(H)** negatively correlated with *ADORA2B* after siRNA transfection. *p < 0.05, **p < 0.01, and ***p < 0.001 are statistically significant.

We found that compared with that in normal lung tissues, the expression level of *ADORA2B* was significantly elevated in tumor tissues of LUAD patients with higher morbidity. To confirm that *ADORA2B* expression is related to LUAD, we investigated the *ADORA2B* gene and protein expression in human LUAD (A549 and NCl-H1299) and normal human bronchial epithelial (HBE) cell lines. Western blotting analysis indicated that the relative ADORA2B protein expression levels in A549 cells and NCl-H1299 cells were significantly higher than those in HBE cells (Figures 8A,B). Next, we examined the mRNA expression level of *ADORA2B* by qRT-PCR and found that the relative *ADORA2B* mRNA expression level was consistent with the protein expression level in the three cell lines (Figure 8C).

As shown in Figure 5C, LinkedOmics was used to identify the differentially expressed genes related to *ADORA2B*. The expression levels of *ADORA2B*-associated genes were positively (*ANXA1*, *ITGA3*, and *S100A6*) or negatively (*KDM2B*, *NEB*, and *CBFA2T2*) correlated with *ADORA2B* expression in LUAD. To verify these results, we measured the mRNA expression levels of these genes. The expression levels of *ANXA1*, *ITGA3*, and *S100A6* were significantly higher in A549 cells than in HBE cells and were positively correlated with *ADORA2B* expression levels (Figure 8D). In contrast, the expression levels of *KDM2B*, *NEB*, and *CBFA2T2* were significantly lower in A549 cells than in HBE cells and negatively correlated with *ADORA2B* expression (Figure 8E). These results were consistent with those from our bioinformatics analyses.

To further confirm these findings, specific ADORA2B siRNAs were transfected into A549 cells to inhibit *ADORA2B* expression. First, we verified the knockdown efficiencies of ADORA2B by qRT-PCR and then selected siRNA-2, which had the highest knockdown efficiency, for subsequent experiments (Figure 8F). Then, we measured the mRNA expression levels of genes that were positively or negatively correlated with *ADORA2B* expression. The expression levels of *ANXA1*, *ITGA3*, and *S100A6* were significantly lower in the *ADORA2B* siRNA group than in the control siRNA group and positively correlated with *ADORA2B* expression (Figure 8G). The expression levels of *KDM2B*, *NEB*, and *CBFA2T2* were significantly higher in the *ADORA2B* siRNA group than in the control siRNA group and negatively correlated with *ADORA2B* expression (Figure 8H). Taken together, *ADORA2B* was highly expressed in human LUAD A549 cells and the expression levels of *ANXA1*, *ITGA3*, and *S100A6* were positively correlated with *ADORA2B* expression, while those of *KDM2B*, *NEB*, and *CBFA2T2* were negatively correlated with *ADORA2B* expression. *In vitro* experimental results were consistent with those of the bioinformatics analyses.

### Knockdown of *ADORA2B* Inhibited the Proliferation and Metastasis of LUAD Cells

**FIGURE 9:**
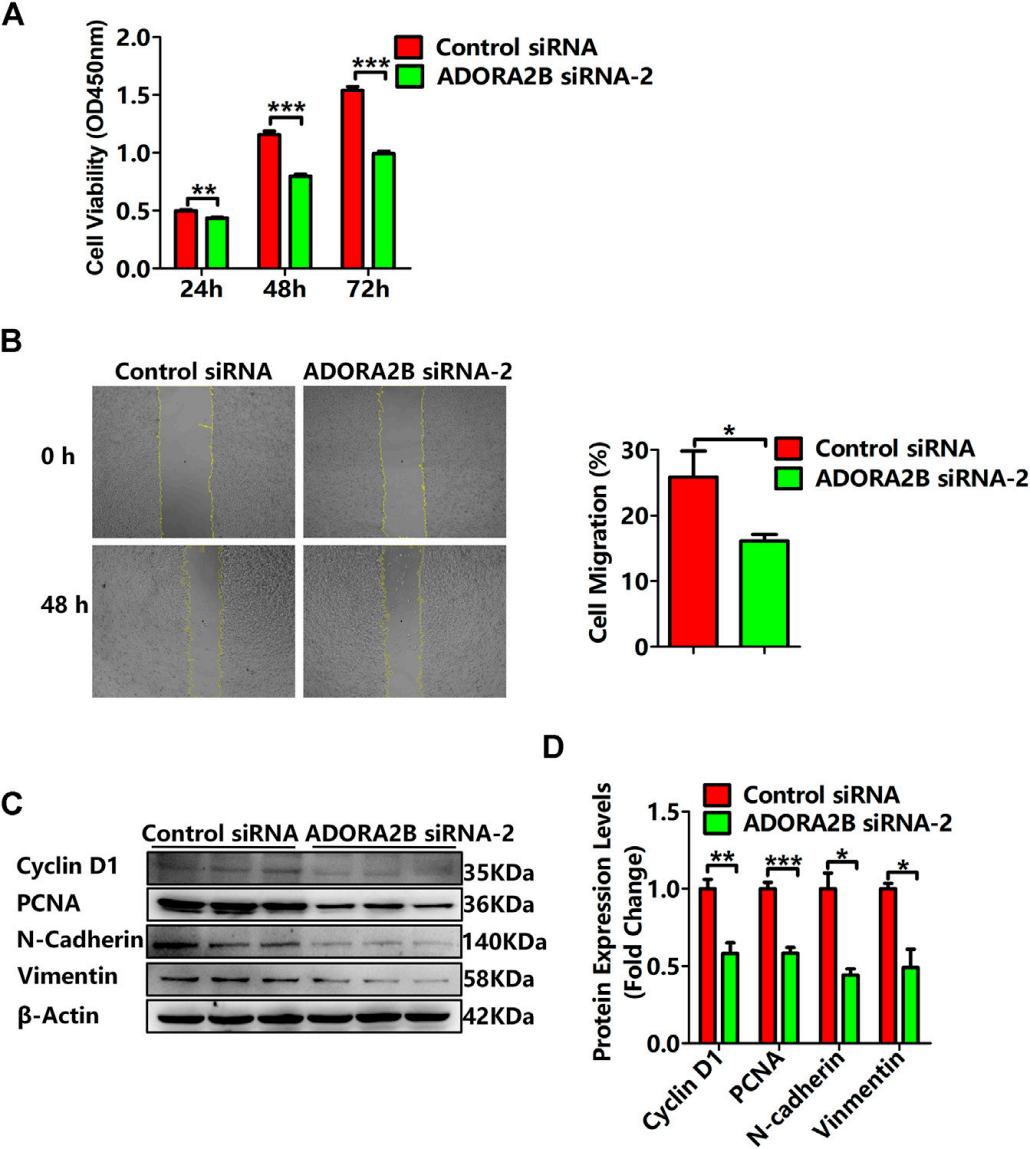
Knockdown of *ADORA2B* inhibited the proliferation and metastasis of LUAD cells. **(A)** The cell viability of LUAD cells transfected with control or *ADORA2B* siRNA was detected by the CCK-8 assay. **(B)** The wound healing assay was used to examine the effect of *ADORA2B* siRNA on A549 cell migration for 48 h (magnification: ×40). **(C)** Relative protein expressions of proliferative (cyclin D1 and PCNA) and metastatic (N-cadherin and vimentin) markers were determined by western blotting. **(D)** Protein quantitative statistics. *p < 0.05, **p < 0.01, and ***p < 0.001 are statistically significant.

Previous studies have demonstrated that *ADORA2B* participates in the proliferation and metastasis of carcinomas, such as prostate and oral cancers (Kasama et al., 2015; Vecchio et al., 2016). To further clarify the role of *ADORA2B* in the progression of LUAD, we performed related functional experiments to verify the function of *ADORA2B*. First, cell proliferation was measured using CCK-8. Compared with that of the control siRNA group, the cell viability of the *ADORA2B* siRNA group was significantly decreased at 24, 48, and 72 h after transfection (Figure 9A). Second, the cell migration rate was evaluated by the wound healing assay, the results of which showed that knockdown of *ADORA2B* reduced the migration capacity of A549 cells after 48 h of induction, partially supporting the role of ADORA2B in promoting metastasis in LUAD (Figure 9B). We further examined the protein expression levels of markers of carcinoma cell proliferation and metastasis after *ADORA2B* knockdown. Western blotting analysis indicated that the relative protein expression levels of proliferative (cyclin D1 and PCNA) and metastatic (N-cadherin and vimentin) markers in the *ADORA2B* siRNA group were significantly lower than those in the control siRNA group (Figures 9C,D). These results demonstrated that knockdown of *ADORA2B* inhibited the proliferation and metastasis of LUAD cells and that *ADORA2B* might participate in the proliferation and metastasis of LUAD cells.

## Discussion

Adenosine is a purinergic molecule that regulates tissue damage and repair. Adenosine receptors belong to the G-protein–coupled receptor superfamily and include A1, A2A, A2B, and A3, which are widely distributed in different human tissues. The expression levels and affinities of adenosine receptors can vary across different tissues and cell types. In addition, these adenosine receptors have different phosphorylation abilities and sensitivities to the G-proteins with which they are conjugated. The A1 receptor is conjugated with the Gi/G0 protein, while the A3 receptor is conjugated with the Gi protein to inhibit the activity of adenylate cyclase and reduce the level of cAMP. In contrast, A2A and A2B receptors are conjugated with Gs proteins to activate the activity of adenylate cyclase and increase the level of cAMP. Low concentrations usually activate the A2A receptor, while higher concentrations of adenosine activate the A2B receptor, which suggests that the basal level of adenosine mainly activates the A2A receptor in a physiological state and that the A2B receptor is activated only when the adenosine level rises above physiological levels or reaches a pathological level (Vecchio et al., 2018; Fenouillet et al., 2019).

Increasing evidence suggests that adenosine receptors are potential diagnostic biomarkers for some diseases, inflammation, and certain types of cancers (Feoktistov and Biaggioni, 2011; Koeppen et al., 2011; Fishman et al., 2012). Additionally, adenosine receptors are upregulated in various tumors (Fishman et al., 2009). *ADORA2B* plays an important role in the occurrence, development, and metastasis of human tumors and is expressed in a variety of tumor cells. In the present study, to observe the functional network of *ADORA2B* in LUAD, we performed bioinformatics analyses to analyze transcriptional sequencing data from clinical sample data obtained from the Gene Expression Omnibus (GEO) and TCGA databases. To determine the role of *ADORA2B* in LUAD, we evaluated the expression pattern of several genes expressed in the disease. The analysis of the Oncomine database revealed fold-changes in gene expression levels that were similar to those reported in previous LUAD studies. According to the mRNA expression level, *ADORA2B* ranked among the top 8–33% of all upregulated genes expressed in LUAD, indicating that *ADORA2B* is significantly overexpressed in LUAD (Figure 1). Survival analysis showed that the OS time (including the overall, one-year, three-year, and five-year survival rates) in the low *ADORA2B* expression group was significantly longer than that in the high *ADORA2B* expression group. These results indicated that a high *ADORA2B* expression predicted a poor prognosis for LUAD patients (Figure 4).

Further analysis using the UALCAN database showed that the mRNA expression levels of *ADORA2B* were significantly higher in LUAD samples than in para-carcinoma tissues. Furthermore, the expression varied depending on the cancer stage, age, smoking habit, nodal metastasis status, and TP53 mutation status. In addition, the copy number of *ADORA2B* was significantly increased in LUAD (Figure 2). These associations indicated the impact of *ADORA2B* expression on human health or the types of biological and social factors that contribute to the pathological process of LUAD.

Cancer progression involves a series of histopathological processes (Xie et al., 2017; Chen et al., 2016) and changes in the functions of specific genes that have potential inhibitory or carcinogenic effects on cancer progression (Cui et al., 2018; Klonowska et al., 2016). Therefore, in the present study, we used the web-based tool, cBioPortal, to detect amplification, mutations, and mRNA expression of *ADORA2B* in LUAD. The major types of *ADORA2B* alterations were mRNA low, deep deletion, and amplification. The fraction of genome altered and mutation type, including not mutated, not profiled for mutations, amplification, gain, diploid, shallow deletion, and deep deletion, were analyzed. These mutations may play key roles in cancer progression and prognosis (Figure 3). Tumorigenesis is an extremely complex process, and new intricate protein–protein interactions and altered signaling pathways accelerate the pathological process of malignant tumors. *ADORA2B* was reported to be involved in tumor-cell proliferation, tumor-cell metastasis, and tumor microenvironment changes (Desmet et al., 2013; Kasama et al., 2015; Vecchio et al., 2016). To further clarify the role of *ADORA2B* in the progression of LUAD, we performed related functional experiments to verify the function of *ADORA2B*. The results indicated that knockdown of *ADORA2B* inhibited the proliferation and metastasis of LUAD cells and that *ADORA2B* might participate in the proliferation and metastasis of LUAD cells (Figure 9).

To identify the functional proteins in LUAD, we used the GeneMANIA web server. Interacting proteins regulate tumor progression. To determine the hierarchy of interacting proteins, we used the LinkedOmics database and drew a heat map of differentially expressed genes correlated with *ADORA2B* expression. In LUAD, *ADORA2B* was predicted to be associated with several *ADORA2B-*associated genes. Among them, *ANXA1* was highly expressed in various malignant tumors (Huang et al., 2015; Okano et al., 2015), and upregulation of ANXA1 levels in the serum was related to the pathological grade and clinical stage of patients with particular forms of LUAD. Knockout of *ANXA1* expression inhibited the proliferation, migration, and invasion of lung carcinoma cells (Fang et al., 2016), while the expression level of *ITGA3* can be used as a diagnostic and prognostic marker for several malignant tumors (Koshizuka et al., 2017; Jiao et al., 2019; Ramovs et al., 2019). *S100A6*, which regulates cytoskeleton protein dynamics, cell proliferation, cell differentiation, calcium metabolism, ubiquitination, and acetylation, was found to promote the proliferation, invasion, migration, and angiogenesis of lung carcinoma cells by inhibiting acetylation of *p53* when overexpressed (Li et al., 2019). KDM2B can reduce the proliferation of carcinoma cells by inhibiting the expression of oncogenes that play important roles in the self-renewal, differentiation, and apoptosis of stem cells (Hong et al., 2016; Goyama and Kitamura, 2017). Therefore, the findings of the present study validated the significance of *ADORA2B* in LUAD (Figure 5). *ADORA2B* interacts with these proteins that regulate important cellular processes associated with cancers and diseases.

Enrichment analysis of target gene sets using GSEA can identify important networks involved in protein targeting, mRNA processing, and transcription factors. ADORA2B was revealed to regulate the FAS signaling pathway, EGF receptor signaling pathway, and apoptosis signaling pathway (Figure 6). The CD73–adenosine receptor signaling pathway can promote the immunosuppressive effect of the tumor microenvironment, which is closely associated with a poor prognosis of multiple tumors (Chen et al., 2020). Immune cell infiltration analysis results suggested that the expression level of *ADORA2B* was associated with infiltrating immune cells in LUAD, including B cells, macrophages, neutrophils, and dendritic cells (Figure 7). These results revealed the indispensable role of *ADORA2B* in the development of LUAD.

The results of our preliminarily bioinformatics analysis suggested that *ADORA2B* can be used as a biomarker for LUAD, and we further confirmed the role of *ADORA2B* in LUAD through *in vitro* experiments. Human LUAD– and bronchial epithelial cell–derived cells lines were cultured *in vitro*, and mRNA and protein levels of *ADORA2B* were analyzed. Our results showed that both the transcription and protein levels of *ADORA2B* were significantly higher in A549 and NCl-H1299 cells than in HBE cells. The expression of genes positively (*ANXA1*, *ITGA3*, and *S100A6*) or negatively (*KDM2B*, *NEB*, and *CBFA2T2*) correlated with *ADORA2B* was consistent with the transcription level of *ADORA2B*. siRNA *ADORA2B* transfection experiments further confirmed these results (Figure 8). Our *in vitro* experiments confirmed the predictive and diagnostic role of ADORA2B in LUAD, but there were some limitations of this study. We tried to obtain human LUAD tissues to verify our *in vitro* results; however, it is difficult to acquire human LUAD tissues owing to ethical and technical reasons. If we get an opportunity to acquire LUAD tissues in the future, we will further investigate the role of *ADORA2B* in LUAD.

## Conclusion

In conclusion, we used several public databases to extract data related to mRNA expression and mutations of *ADORA2B* and to the expression of genes associated with *ADORA2B* in LUAD. Furthermore, we predicted the differentially expressed genes correlated with *ADORA2B* expression in LUAD. Survival analysis showed that the OS time in the low *ADORA2B* expression group was longer than that in the high *ADORA2B* expression group. These results indicated that a high expression of *ADORA2B* predicted a poor prognosis for LUAD patients. Enrichment analysis of target gene sets using GSEA revealed important networks of the *ADORA2B* signaling pathway. Immune infiltration analysis results suggested that the expression level of *ADORA2B* was associated with immune cell infiltration in LUAD. *In vitro* experiments revealed that the relative *ADORA2B* expression levels in LUAD cells were significantly higher than those in normal bronchial epithelial cells. Knockdown of *ADORA2B* inhibited the proliferation and metastasis of LUAD cells, indicating that *ADORA2B* might participate in the proliferation and metastasis of LUAD cells. Taken together, our results revealed the significance of *ADORA2B* expression and its interaction networks in LUAD; the results of our *in vitro* experiments were consistent with those of the bioinformatics analysis. Although A549, NCl-H1299, and HBE cell lines were mainly used in the current experiments, we will verify these results with human and murine LUAD tissues in future studies. Our findings provide valuable insights into *ADORA2B* as a potential diagnostic and prognostic marker for LUAD and thus provide a foundation for further research focusing on LUAD biomarker development.

## Data Availability

Okayama Lung data were downloaded from the Oncomine database under Series accession number GSE31210 (http://www.ncbi.nlm.nih.gov/geo/query/acc.cgi?acc=GSE31210). Landi Lung data were downloaded from the Oncomine database under Series accession number GSE10072 (http://www.ncbi.nlm.nih.gov/geo/query/acc.cgi?acc=GSE10072). Selamat Lung data were downloaded from the Oncomine database under Series accession number GSE32863 (http://www.ncbi.nlm.nih.gov/geo/query/acc.cgi?acc=GSE32863). Stearman Lung data were downloaded from the Oncomine database under Series accession number GSE2514 (http://www.ncbi.nlm.nih.gov/geo/query/acc.cgi?acc=GSE2514). Beer Lung data were downloaded from the Oncomine database under the Array Type: HumanGeneFL Array, Measured 5,338 genes, 7,133 reporters (Beer, D. G., Kardia, S. L., Huang, C. C., Giordano, T. J., Levin, A. M., Misek, D. E., et al., 2002). Gene expression profiles predict survival of patients with lung adenocarcinoma (Nat. Med. 8, 816–824. doi: 10.1038/nm733). Su Lung data were downloaded from the Oncomine database under Series accession number GSE7670 (http://www.ncbi.nlm.nih.gov/geo/query/acc.cgi?acc=GSE7670). Bhattacharjee Lung data were downloaded from the Oncomine database under the Array Type: Human Genome U95A-Av2 Array, Measured 8,603 genes, 12,651 reporters (Bhattacharjee, A., Richards, W. G., Staunton, J., Li, C., Monti, S., Vasa, P., et al., 2001). Classification of human lung carcinomas by mRNA expression profiling reveals distinct adenocarcinoma subclasses (Proc. Natl. Acad. Sci. U S A98, 13790–13795. doi: 10.1073/pnas.191502998). Garber Lung data were downloaded from the Oncomine database under Series accession number GSE3398 (http://www.ncbi.nlm.nih.gov/geo/query/acc.cgi?acc=GSE3398). OncoPrint data were downloaded from the cBioPortal database Shortened URL https://bit.ly/3oTsOd5. Mutation data were downloaded from the cBioPortal database Shortened URL https://bit.ly/2ObTF7t. Clinical attribute data were downloaded from the cBioPortal database Shortened URL https://bit.ly/3rnelHU. LinkedOmics data were downloaded from the LinkedOmics database under ID number ID-62189.

## Abbreviations

AAMP, angio-associated migratory cell protein; ADORA2B, adenosine receptor A2B; ALK, anaplastic lymphoma kinase; ANXA1, annexin A1; CBFA2T2, CBFA2/RUNX1 partner transcriptional co-repressor 2; BRAF, V-raf murine sarcoma viral oncogene homolog B1; DCC, netrin 1 receptor; DPH, diphthamide biosynthesis 1; EGFR, epidermal growth factor receptor; GEO, Gene Expression Omnibus; HER2, human epidermal growth factor receptor 2; ITGA3, integrin subunit alpha 3; KCNJ3, potassium inwardly rectifying channel subfamily J member 3; KDM2B, lysine demethylase 2B; KRAS, Kirsten rat sarcoma viral oncogene; LUAD, lung adenocarcinoma; NEB, nebulin; OS, overall survival; PEX6, peroxisomal biogenesis factor 6; RET, rearranged during transfection; ROS1, ROS proto-oncogene 1; SLC29A2, solute carrier family 29 member 2; SLC9A3R2, solute carrier family 9 member A3 regulator 2; S100A6, S100 calcium binding protein A6; TCGA, The Cancer Genome Atlas; TPD52L2, tumor protein D52-like 2; ZNF267, zinc finger protein 267.
