# Supplementary material for: Expression and Gene Regulation Network of Adenosine Receptor A2B in Lung Adenocarcinoma: A Potential Diagnostic and Prognostic Biomarker
Source: Front Mol Biosci. 2021 Jul 19;8:663011. doi: 10.3389/fmolb.2021.663011 (PMC8326519; doi:10.3389/fmolb.2021.663011)
Supplement: Supplementary file 1 [file Table1.DOCX]

Supplementary Table 1. Related primer sequences

| Genes |  | Sequences |
| --- | --- | --- |
| β-actin | F(5’- 3’) | GCCCTGAGGCACTCTTCCA |
|  | R(5’- 3’) | GAAGGTAGTTTCGTGGATGCCA |
| ANXA1 | F(5’- 3’) | GCGGTGAGCCCCTATCCTA |
|  | R(5’- 3’) | TGATGGTTGCTTCATCCACAC |
| ITGA3 | F(5’- 3’) | TCAACCTGGATACCCGATTCC |
|  | R(5’- 3’) | GCTCTGTCTGCCGATGGAG |
| S100A6 | F(5’- 3’) | GGGAGGGTGACAAGCACAC |
|  | R(5’- 3’) | AGCTTCGAGCCAATGGTGAG |
| KDM2B | F(5’- 3’) | GGGTTCCCCTGATATTTCGAGA |
|  | R(5’- 3’) | GCTCCCCACTAGGAGTTTGAC |
| NEBULIN | F(5’- 3’) | GGAGGCCGCCTTTTCAGAA |
|  | R(5’- 3’) | GGAAGGATCAGGATACGGAAAGT |
| CBFA2T2 | F(5’- 3’) | CCAGTGTCCTTCACTCCTACT |
|  | R(5’- 3’) | TTGCTGAGTTGTCGAGCACC |
| Control si-RNA |  | UUCUCCGAACGUGUCACGUTT |
|  |  | ACGUGACACGUUCGGAGAATT |
| ADORA2B si-RNA-1 |  | ACUUCUACGGCUGCCUCUUTT |
|  |  | AAGAGGCAGCCGUAGAAGUTT |
| ADORA2B si-RNA-2 |  | GGAUGGAACCACGAAUGAATT |
|  |  | UUCAUUCGUGGUUCCAUCCTT |
| ADORA2B si-RNA-3 |  | CCAAGUGGGCAAUGAAUAUTT |
|  |  | AUAUUCAUUGCCCACUUGGTT |
