# Supplementary material for: Expression and Gene Regulation Network of Adenosine Receptor A2B in Lung Adenocarcinoma: A Potential Diagnostic and Prognostic Biomarker
Source: Front Mol Biosci. 2021 Jul 19;8:663011. doi: 10.3389/fmolb.2021.663011 (PMC8326519; doi:10.3389/fmolb.2021.663011)
Supplement: Supplementary file 3 [file Table2.DOCX]

Supplementary Table 2. Patient order in the Oncoprint

|  |
| --- |
| TCGA-44-A4SS |
| TCGA-44-7667 |
| TCGA-86-8054 |
| TCGA-44-2665 |
| TCGA-44-3396 |
| TCGA-44-7661 |
| TCGA-55-A48Z |
| TCGA-75-6206 |
| TCGA-05-5428 |
| TCGA-38-4625 |
| TCGA-44-2666 |
| TCGA-44-3918 |
| TCGA-49-4494 |
| TCGA-50-5930 |
| TCGA-55-A4DG |
| TCGA-55-6986 |
| TCGA-55-7725 |
| TCGA-55-7907 |
| TCGA-64-1680 |
| TCGA-69-8254 |
| TCGA-73-4662 |
| TCGA-73-4666 |
| TCGA-73-4677 |
| TCGA-L9-A5IP |
| TCGA-78-7145 |
| TCGA-NJ-A7XG |
| TCGA-80-5607 |
| TCGA-86-7714 |
| TCGA-91-A4BD |
| TCGA-91-8496 |
| TCGA-4B-A93V |
| TCGA-05-4244 |
| TCGA-05-4249 |
| TCGA-05-4250 |
| TCGA-05-4382 |
| TCGA-05-4384 |
| TCGA-05-4389 |
| TCGA-05-4390 |
| TCGA-05-4395 |
| TCGA-05-4396 |
| TCGA-05-4397 |
| TCGA-05-4398 |
| TCGA-05-4402 |
| TCGA-05-4403 |
| TCGA-05-4405 |
| TCGA-05-4410 |
| TCGA-05-4415 |
| TCGA-05-4417 |
| TCGA-05-4418 |
| TCGA-05-4420 |
| TCGA-05-4422 |
| TCGA-05-4424 |
| TCGA-05-4425 |
| TCGA-05-4426 |
| TCGA-05-4427 |
| TCGA-05-4430 |
| TCGA-05-4432 |
| TCGA-05-4433 |
| TCGA-05-4434 |
| TCGA-05-5423 |
| TCGA-05-5425 |
| TCGA-05-5429 |
| TCGA-05-5715 |
| TCGA-35-3615 |
| TCGA-35-4122 |
| TCGA-35-4123 |
| TCGA-35-5375 |
| TCGA-38-A44F |
| TCGA-38-4626 |
| TCGA-38-4627 |
| TCGA-38-4628 |
| TCGA-38-4631 |
| TCGA-38-4632 |
| TCGA-38-6178 |
| TCGA-38-7271 |
| TCGA-44-A4SU |
| TCGA-44-A47A |
| TCGA-44-A47B |
| TCGA-44-A47G |
| TCGA-44-A479 |
| TCGA-44-2655 |
| TCGA-44-2656 |
| TCGA-44-2657 |
| TCGA-44-2659 |
| TCGA-44-2662 |
| TCGA-44-2668 |
| TCGA-44-3398 |
| TCGA-44-3919 |
| TCGA-44-4112 |
| TCGA-44-5643 |
| TCGA-44-5644 |
| TCGA-44-5645 |
| TCGA-44-6145 |
| TCGA-44-6146 |
| TCGA-44-6148 |
| TCGA-44-6774 |
| TCGA-44-6775 |
| TCGA-44-6776 |
| TCGA-44-6777 |
| TCGA-44-6778 |
| TCGA-44-6779 |
| TCGA-44-7659 |
| TCGA-44-7660 |
| TCGA-44-7662 |
| TCGA-44-7669 |
| TCGA-44-7670 |
| TCGA-44-7671 |
| TCGA-44-7672 |
| TCGA-44-8117 |
| TCGA-44-8119 |
| TCGA-44-8120 |
| TCGA-49-AAQV |
| TCGA-49-AAR0 |
| TCGA-49-AAR2 |
| TCGA-49-AAR3 |
| TCGA-49-AAR4 |
| TCGA-49-AAR9 |
| TCGA-49-AARE |
| TCGA-49-AARN |
| TCGA-49-AARO |
| TCGA-49-AARQ |
| TCGA-49-AARR |
| TCGA-49-4486 |
| TCGA-49-4487 |
| TCGA-49-4488 |
| TCGA-49-4490 |
| TCGA-49-4501 |
| TCGA-49-4505 |
| TCGA-49-4506 |
| TCGA-49-4507 |
| TCGA-49-4510 |
| TCGA-49-4512 |
| TCGA-49-4514 |
| TCGA-49-6742 |
| TCGA-49-6743 |
| TCGA-49-6744 |
| TCGA-49-6745 |
| TCGA-49-6761 |
| TCGA-49-6767 |
| TCGA-50-5044 |
| TCGA-50-5045 |
| TCGA-50-5049 |
| TCGA-50-5051 |
| TCGA-50-5055 |
| TCGA-50-5066 |
| TCGA-50-5068 |
| TCGA-50-5072 |
| TCGA-50-5931 |
| TCGA-50-5932 |
| TCGA-50-5933 |
| TCGA-50-5935 |
| TCGA-50-5936 |
| TCGA-50-5939 |
| TCGA-50-5941 |
| TCGA-50-5942 |
| TCGA-50-5944 |
| TCGA-50-5946 |
| TCGA-50-6590 |
| TCGA-50-6591 |
| TCGA-50-6592 |
| TCGA-50-6593 |
| TCGA-50-6594 |
| TCGA-50-6595 |
| TCGA-50-6597 |
| TCGA-50-6673 |
| TCGA-50-7109 |
| TCGA-50-8457 |
| TCGA-50-8459 |
| TCGA-50-8460 |
| TCGA-53-A4EZ |
| TCGA-53-7624 |
| TCGA-53-7626 |
| TCGA-53-7813 |
| TCGA-55-A4DF |
| TCGA-55-A48X |
| TCGA-55-A48Y |
| TCGA-55-A57B |
| TCGA-55-A490 |
| TCGA-55-A491 |
| TCGA-55-A492 |
| TCGA-55-A493 |
| TCGA-55-A494 |
| TCGA-55-1592 |
| TCGA-55-1594 |
| TCGA-55-1595 |
| TCGA-55-1596 |
| TCGA-55-5899 |
| TCGA-55-6543 |
| TCGA-55-6642 |
| TCGA-55-6712 |
| TCGA-55-6968 |
| TCGA-55-6969 |
| TCGA-55-6970 |
| TCGA-55-6971 |
| TCGA-55-6972 |
| TCGA-55-6975 |
| TCGA-55-6978 |
| TCGA-55-6979 |
| TCGA-55-6980 |
| TCGA-55-6981 |
| TCGA-55-6982 |
| TCGA-55-6983 |
| TCGA-55-6984 |
| TCGA-55-6985 |
| TCGA-55-6987 |
| TCGA-55-7281 |
| TCGA-55-7283 |
| TCGA-55-7284 |
| TCGA-55-7570 |
| TCGA-55-7573 |
| TCGA-55-7574 |
| TCGA-55-7576 |
| TCGA-55-7724 |
| TCGA-55-7726 |
| TCGA-55-7727 |
| TCGA-55-7728 |
| TCGA-55-7815 |
| TCGA-55-7903 |
| TCGA-55-7910 |
| TCGA-55-7911 |
| TCGA-55-7913 |
| TCGA-55-7914 |
| TCGA-55-7994 |
| TCGA-55-7995 |
| TCGA-55-8085 |
| TCGA-55-8087 |
| TCGA-55-8089 |
| TCGA-55-8090 |
| TCGA-55-8091 |
| TCGA-55-8092 |
| TCGA-55-8094 |
| TCGA-55-8096 |
| TCGA-55-8097 |
| TCGA-55-8203 |
| TCGA-55-8204 |
| TCGA-55-8205 |
| TCGA-55-8206 |
| TCGA-55-8207 |
| TCGA-55-8208 |
| TCGA-55-8299 |
| TCGA-55-8301 |
| TCGA-55-8302 |
| TCGA-55-8505 |
| TCGA-55-8506 |
| TCGA-55-8507 |
| TCGA-55-8508 |
| TCGA-55-8510 |
| TCGA-55-8511 |
| TCGA-55-8512 |
| TCGA-55-8513 |
| TCGA-55-8514 |
| TCGA-55-8614 |
| TCGA-55-8615 |
| TCGA-55-8616 |
| TCGA-55-8619 |
| TCGA-55-8620 |
| TCGA-55-8621 |
| TCGA-62-A46O |
| TCGA-62-A46P |
| TCGA-62-A46R |
| TCGA-62-A46S |
| TCGA-62-A46U |
| TCGA-62-A46V |
| TCGA-62-A46Y |
| TCGA-62-A470 |
| TCGA-62-A471 |
| TCGA-62-A472 |
| TCGA-62-8394 |
| TCGA-62-8395 |
| TCGA-62-8397 |
| TCGA-62-8398 |
| TCGA-62-8399 |
| TCGA-62-8402 |
| TCGA-64-1676 |
| TCGA-64-1677 |
| TCGA-64-1679 |
| TCGA-64-1681 |
| TCGA-64-5774 |
| TCGA-64-5775 |
| TCGA-64-5778 |
| TCGA-64-5779 |
| TCGA-64-5781 |
| TCGA-64-5815 |
| TCGA-67-3770 |
| TCGA-67-3771 |
| TCGA-67-3772 |
| TCGA-67-3773 |
| TCGA-67-3774 |
| TCGA-67-4679 |
| TCGA-67-6215 |
| TCGA-67-6216 |
| TCGA-67-6217 |
| TCGA-69-A59K |
| TCGA-69-7760 |
| TCGA-69-7761 |
| TCGA-69-7763 |
| TCGA-69-7764 |
| TCGA-69-7765 |
| TCGA-69-7973 |
| TCGA-69-7974 |
| TCGA-69-7978 |
| TCGA-69-7979 |
| TCGA-69-7980 |
| TCGA-69-8253 |
| TCGA-69-8255 |
| TCGA-71-6725 |
| TCGA-73-A9RS |
| TCGA-73-4658 |
| TCGA-73-4659 |
| TCGA-73-4668 |
| TCGA-73-4670 |
| TCGA-73-4675 |
| TCGA-73-4676 |
| TCGA-73-7498 |
| TCGA-73-7499 |
| TCGA-J2-A4AD |
| TCGA-J2-A4AE |
| TCGA-J2-A4AG |
| TCGA-J2-8192 |
| TCGA-J2-8194 |
| TCGA-75-5122 |
| TCGA-75-5125 |
| TCGA-75-5126 |
| TCGA-75-5146 |
| TCGA-75-5147 |
| TCGA-75-6203 |
| TCGA-75-6205 |
| TCGA-75-6207 |
| TCGA-75-6211 |
| TCGA-75-6212 |
| TCGA-75-6214 |
| TCGA-75-7025 |
| TCGA-75-7027 |
| TCGA-75-7030 |
| TCGA-75-7031 |
| TCGA-L4-A4E5 |
| TCGA-L4-A4E6 |
| TCGA-L9-A7SV |
| TCGA-L9-A8F4 |
| TCGA-L9-A50W |
| TCGA-L9-A443 |
| TCGA-L9-A444 |
| TCGA-L9-A743 |
| TCGA-MN-A4N1 |
| TCGA-MN-A4N4 |
| TCGA-MN-A4N5 |
| TCGA-MP-A4SV |
| TCGA-MP-A4SW |
| TCGA-MP-A4SY |
| TCGA-MP-A4T4 |
| TCGA-MP-A4T6 |
| TCGA-MP-A4T7 |
| TCGA-MP-A4T8 |
| TCGA-MP-A4T9 |
| TCGA-MP-A4TA |
| TCGA-MP-A4TC |
| TCGA-MP-A4TD |
| TCGA-MP-A4TE |
| TCGA-MP-A4TF |
| TCGA-MP-A4TH |
| TCGA-MP-A4TI |
| TCGA-MP-A4TJ |
| TCGA-MP-A4TK |
| TCGA-MP-A5C7 |
| TCGA-78-7146 |
| TCGA-78-7147 |
| TCGA-78-7148 |
| TCGA-78-7149 |
| TCGA-78-7150 |
| TCGA-78-7152 |
| TCGA-78-7153 |
| TCGA-78-7154 |
| TCGA-78-7155 |
| TCGA-78-7156 |
| TCGA-78-7158 |
| TCGA-78-7159 |
| TCGA-78-7160 |
| TCGA-78-7161 |
| TCGA-78-7162 |
| TCGA-78-7163 |
| TCGA-78-7166 |
| TCGA-78-7167 |
| TCGA-78-7220 |
| TCGA-78-7535 |
| TCGA-78-7536 |
| TCGA-78-7537 |
| TCGA-78-7539 |
| TCGA-78-7540 |
| TCGA-78-7542 |
| TCGA-78-7633 |
| TCGA-78-8640 |
| TCGA-78-8648 |
| TCGA-78-8655 |
| TCGA-78-8660 |
| TCGA-78-8662 |
| TCGA-NJ-A4YF |
| TCGA-NJ-A4YG |
| TCGA-NJ-A4YI |
| TCGA-NJ-A4YP |
| TCGA-NJ-A4YQ |
| TCGA-NJ-A55A |
| TCGA-NJ-A55O |
| TCGA-NJ-A55R |
| TCGA-O1-A52J |
| TCGA-80-5608 |
| TCGA-80-5611 |
| TCGA-S2-AA1A |
| TCGA-83-5908 |
| TCGA-86-A4JF |
| TCGA-86-A4P7 |
| TCGA-86-A4P8 |
| TCGA-86-A456 |
| TCGA-86-6562 |
| TCGA-86-6851 |
| TCGA-86-7701 |
| TCGA-86-7711 |
| TCGA-86-7713 |
| TCGA-86-7953 |
| TCGA-86-7954 |
| TCGA-86-7955 |
| TCGA-86-8055 |
| TCGA-86-8056 |
| TCGA-86-8073 |
| TCGA-86-8074 |
| TCGA-86-8075 |
| TCGA-86-8076 |
| TCGA-86-8278 |
| TCGA-86-8279 |
| TCGA-86-8280 |
| TCGA-86-8281 |
| TCGA-86-8358 |
| TCGA-86-8359 |
| TCGA-86-8585 |
| TCGA-86-8668 |
| TCGA-86-8669 |
| TCGA-86-8671 |
| TCGA-86-8672 |
| TCGA-86-8673 |
| TCGA-86-8674 |
| TCGA-91-A4BC |
| TCGA-91-6828 |
| TCGA-91-6829 |
| TCGA-91-6830 |
| TCGA-91-6831 |
| TCGA-91-6835 |
| TCGA-91-6836 |
| TCGA-91-6840 |
| TCGA-91-6847 |
| TCGA-91-6848 |
| TCGA-91-6849 |
| TCGA-91-7771 |
| TCGA-91-8497 |
| TCGA-91-8499 |
| TCGA-93-A4JN |
| TCGA-93-A4JO |
| TCGA-93-A4JP |
| TCGA-93-A4JQ |
| TCGA-93-7347 |
| TCGA-93-7348 |
| TCGA-93-8067 |
| TCGA-95-A4VK |
| TCGA-95-A4VN |
| TCGA-95-A4VP |
| TCGA-95-7039 |
| TCGA-95-7043 |
| TCGA-95-7562 |
| TCGA-95-7567 |
| TCGA-95-7944 |
| TCGA-95-7947 |
| TCGA-95-7948 |
| TCGA-95-8039 |
| TCGA-95-8494 |
| TCGA-97-A4LX |
| TCGA-97-A4M0 |
| TCGA-97-A4M1 |
| TCGA-97-A4M2 |
| TCGA-97-A4M3 |
| TCGA-97-A4M5 |
| TCGA-97-A4M6 |
| TCGA-97-A4M7 |
| TCGA-97-7546 |
| TCGA-97-7547 |
| TCGA-97-7552 |
| TCGA-97-7553 |
| TCGA-97-7554 |
| TCGA-97-7937 |
| TCGA-97-7938 |
| TCGA-97-7941 |
| TCGA-97-8171 |
| TCGA-97-8172 |
| TCGA-97-8174 |
| TCGA-97-8175 |
| TCGA-97-8176 |
| TCGA-97-8177 |
| TCGA-97-8179 |
| TCGA-97-8547 |
| TCGA-97-8552 |
| TCGA-99-AA5R |
| TCGA-99-7458 |
| TCGA-99-8025 |
| TCGA-99-8028 |
| TCGA-99-8032 |
| TCGA-99-8033 |
