# Supplementary material for: Expression and Gene Regulation Network of Adenosine Receptor A2B in Lung Adenocarcinoma: A Potential Diagnostic and Prognostic Biomarker
Source: Front Mol Biosci. 2021 Jul 19;8:663011. doi: 10.3389/fmolb.2021.663011 (PMC8326519; doi:10.3389/fmolb.2021.663011)
Supplement: Supplementary file 4 [file Table5.DOCX]

**7A-ADORA2B (HBE&A549)**


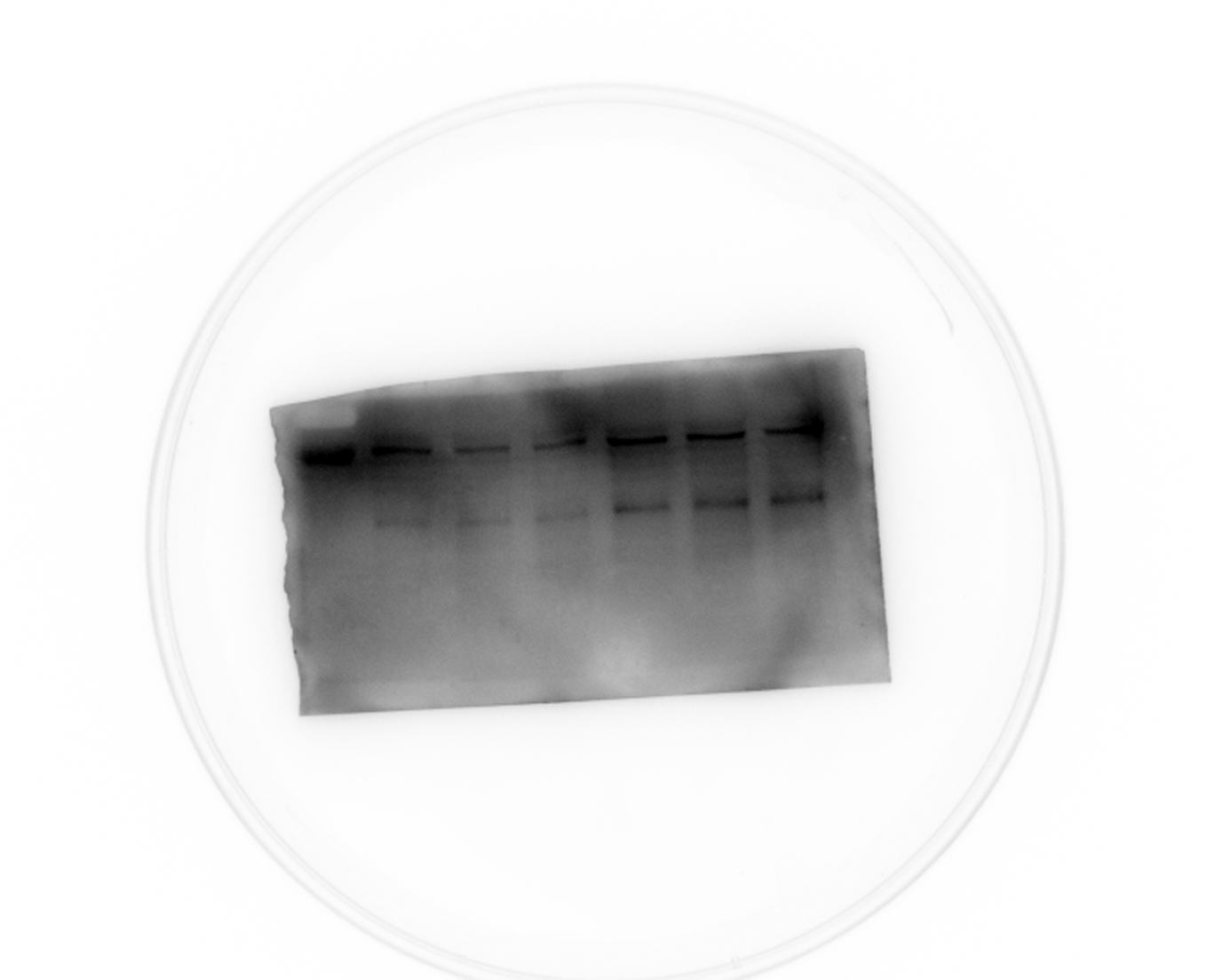


**7A-β-Actin (HBE&A549)**

**
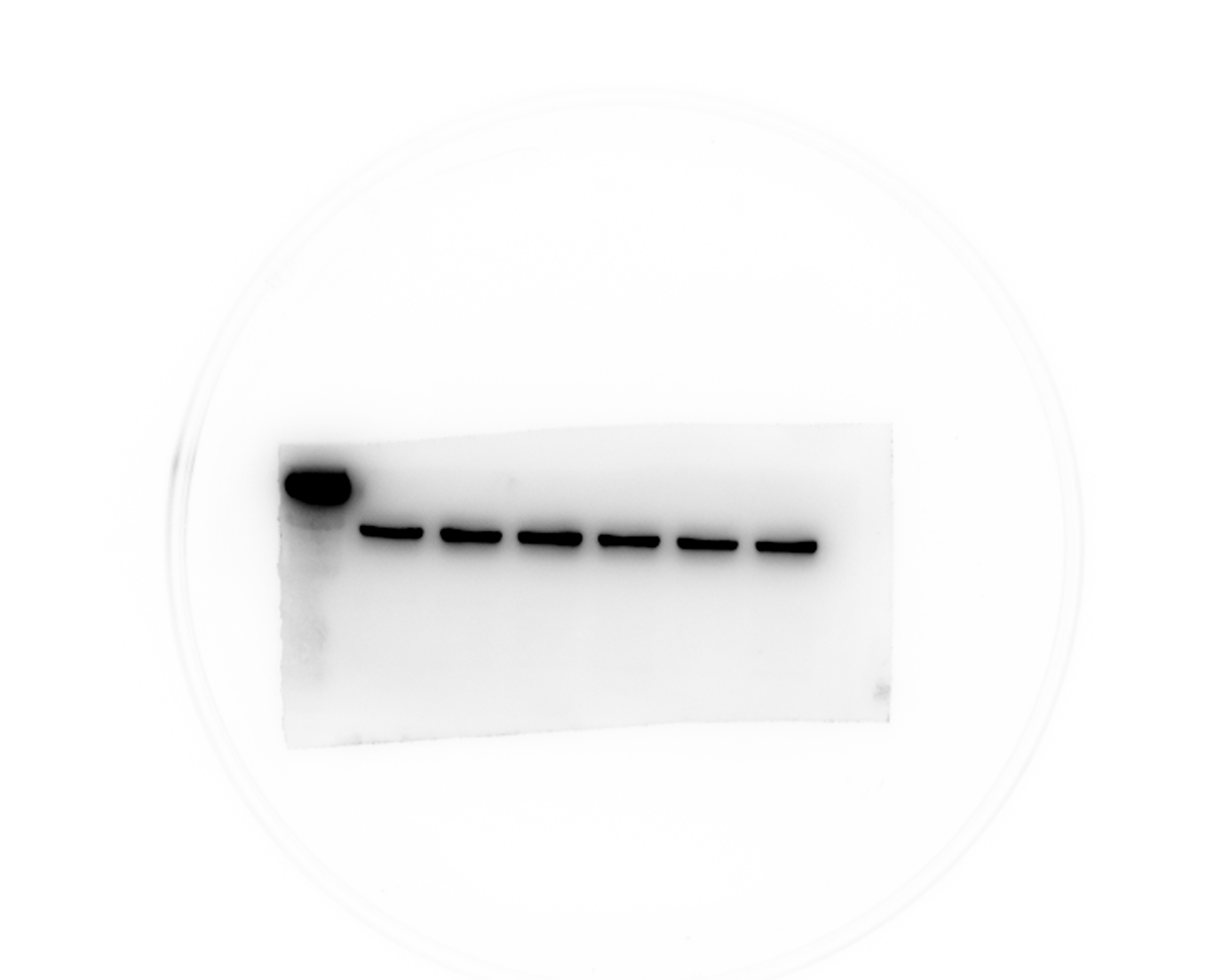
**

**7A-ADORA2B (HBE&H1299)**

**
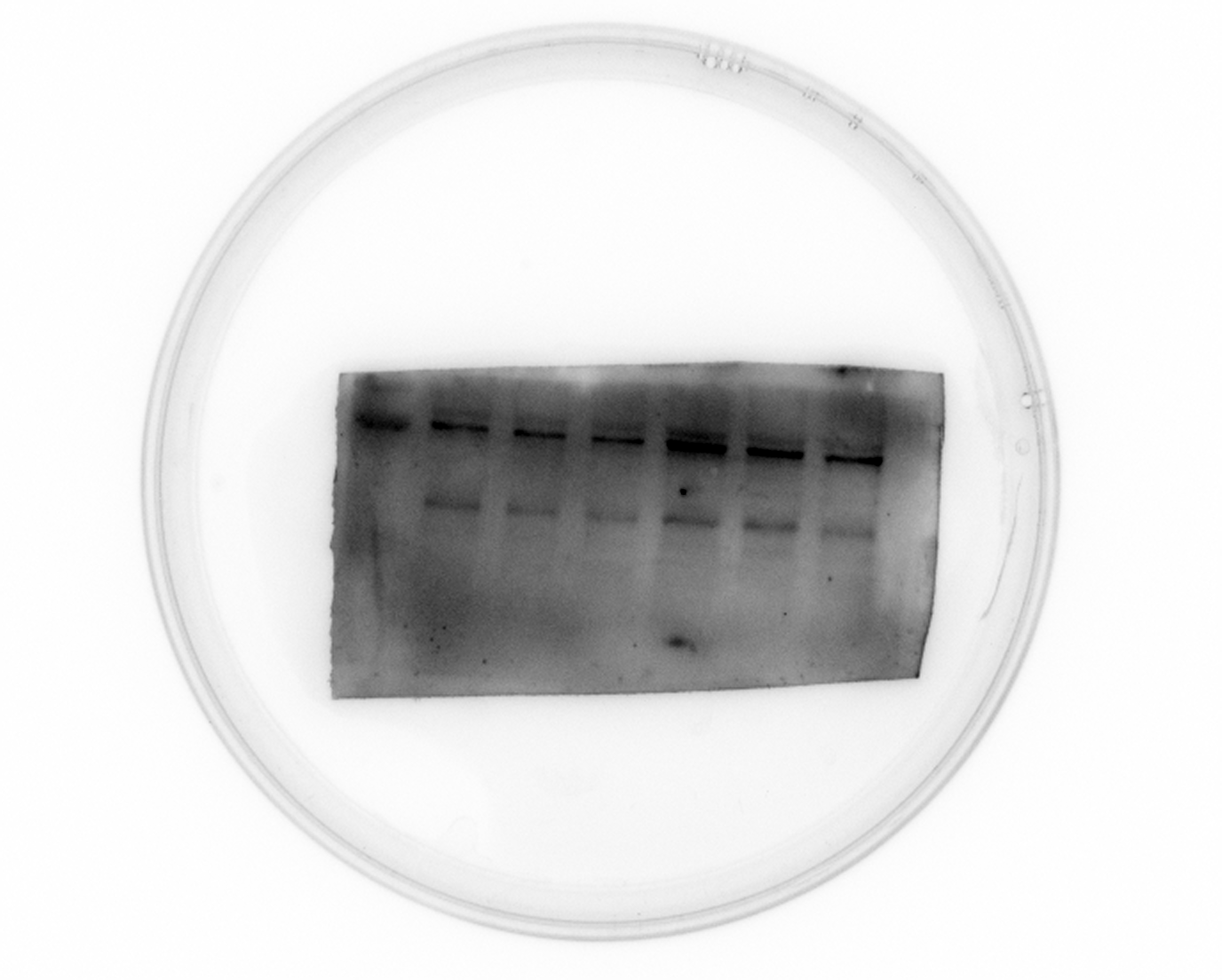
**

**7A-β-Actin (HBE&H1299)**

**
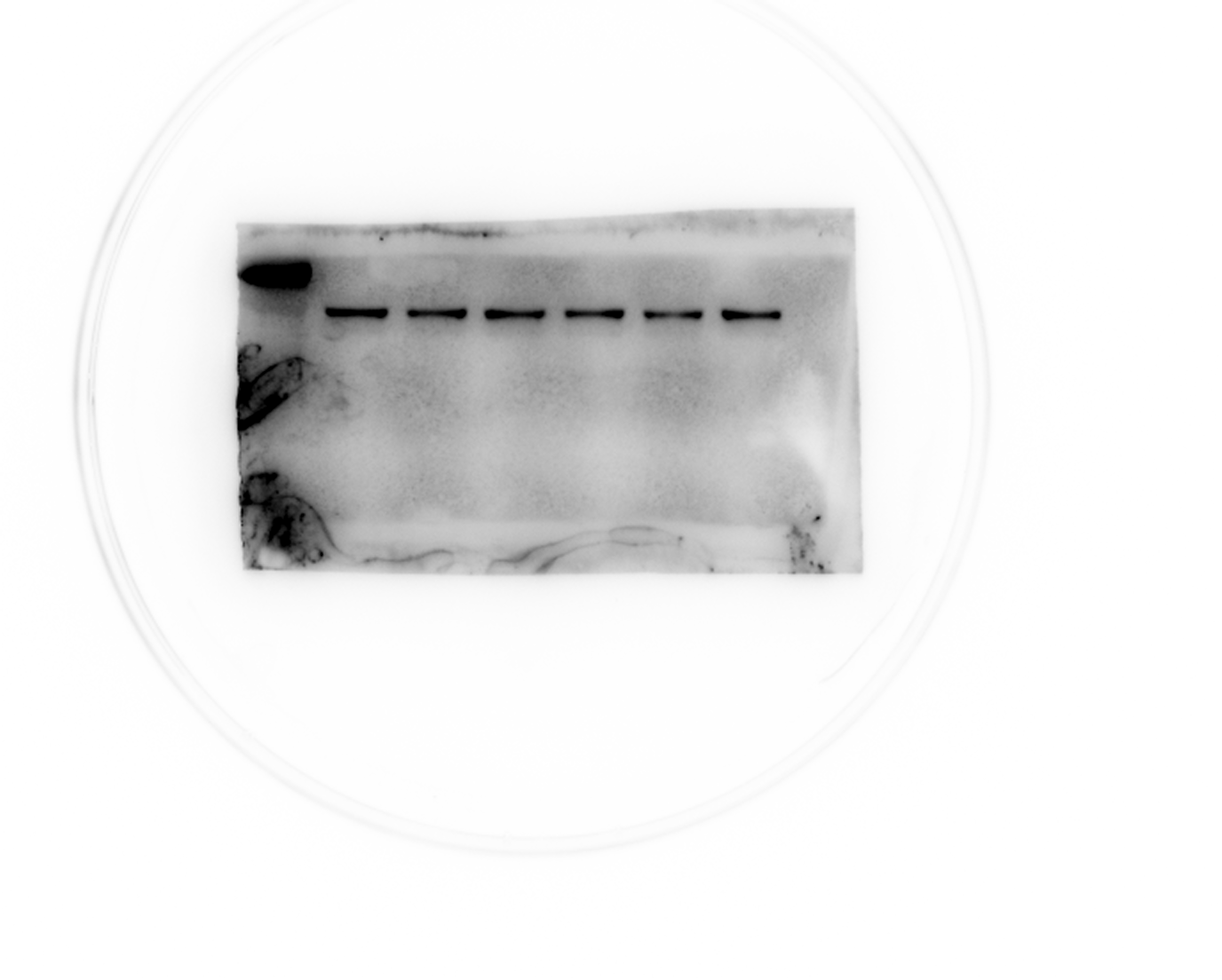
**

**8C-β-Actin**

**
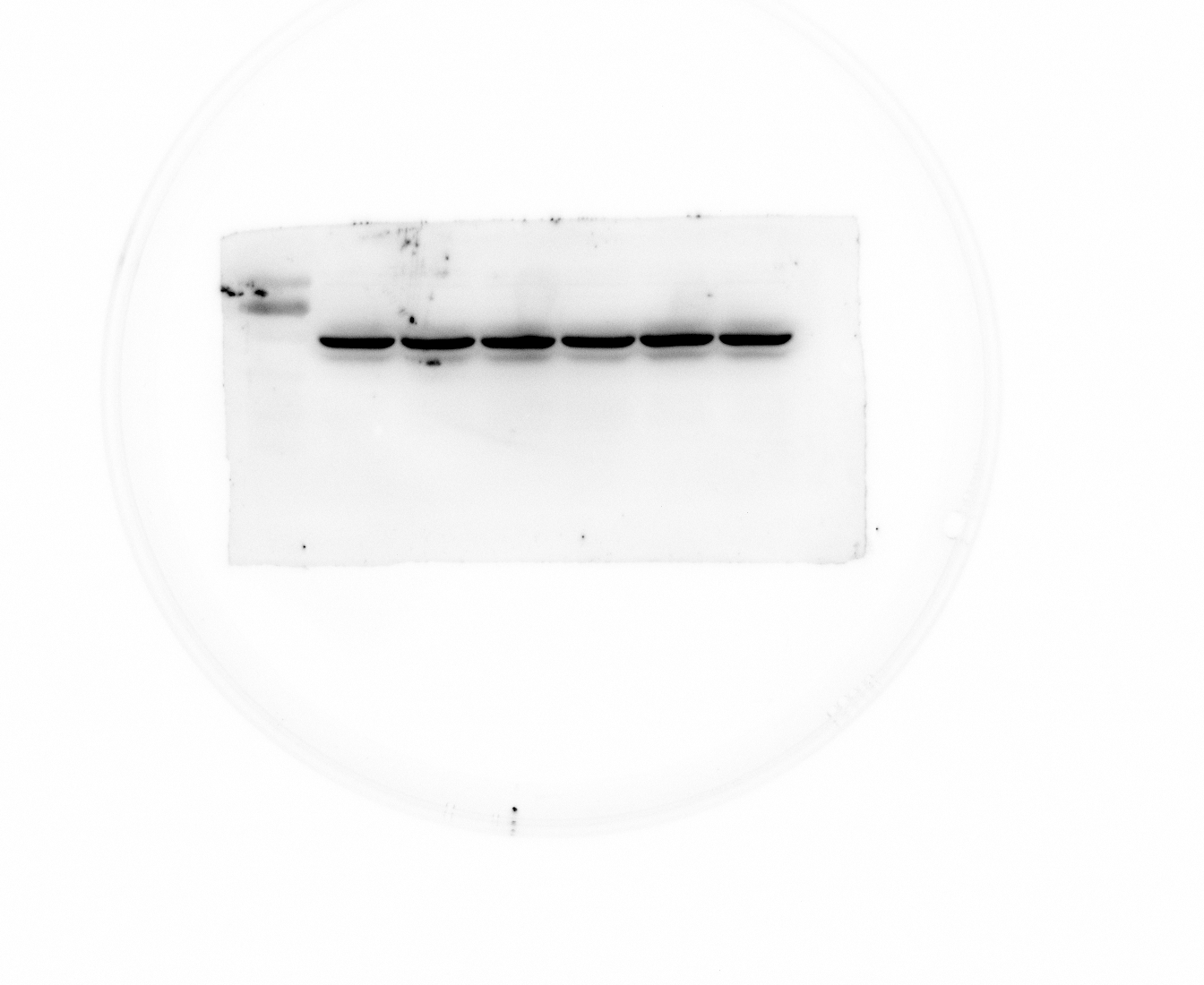
**

**8C-Cyclin D1**

**
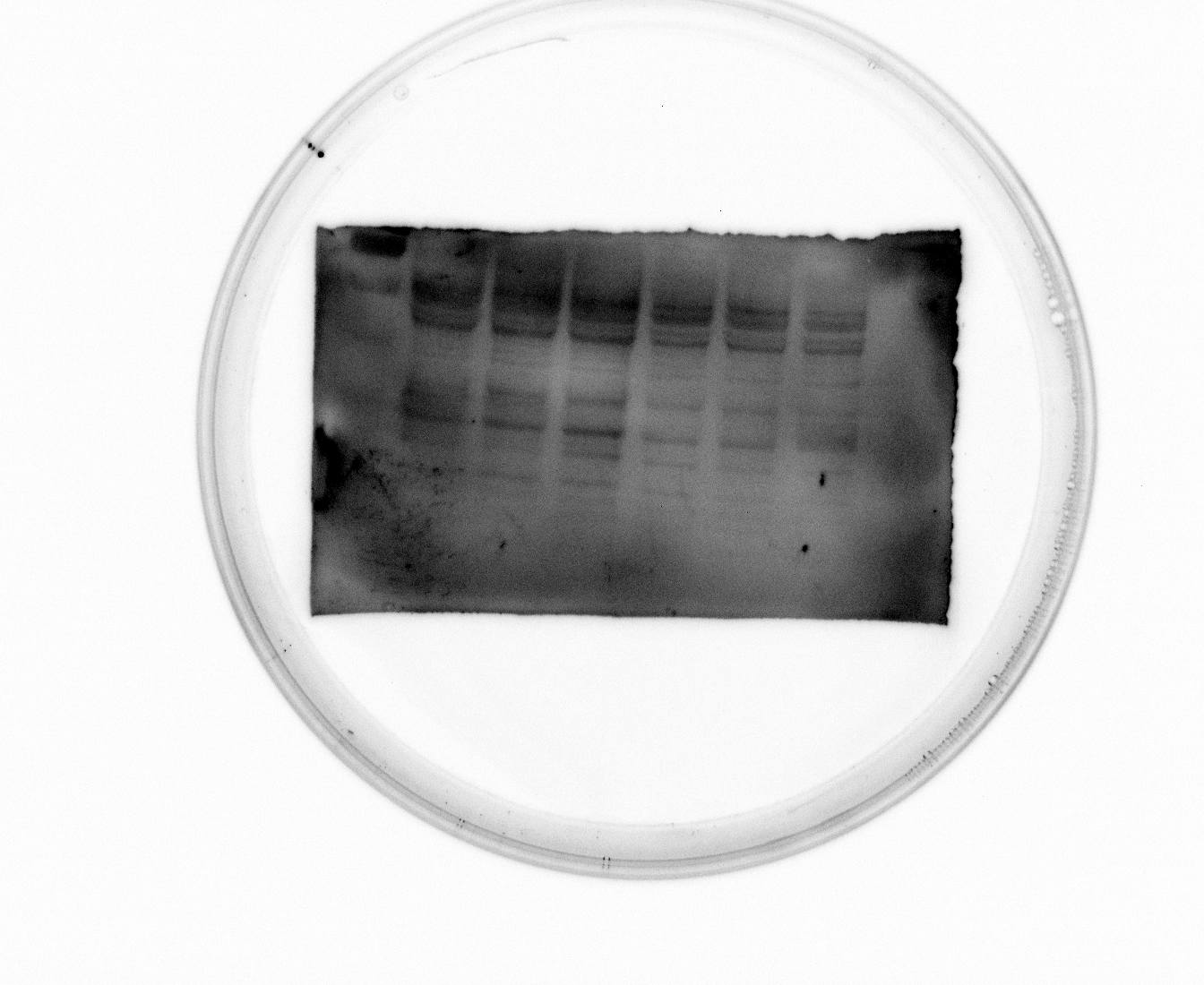
**

**8C-PCNA**

**
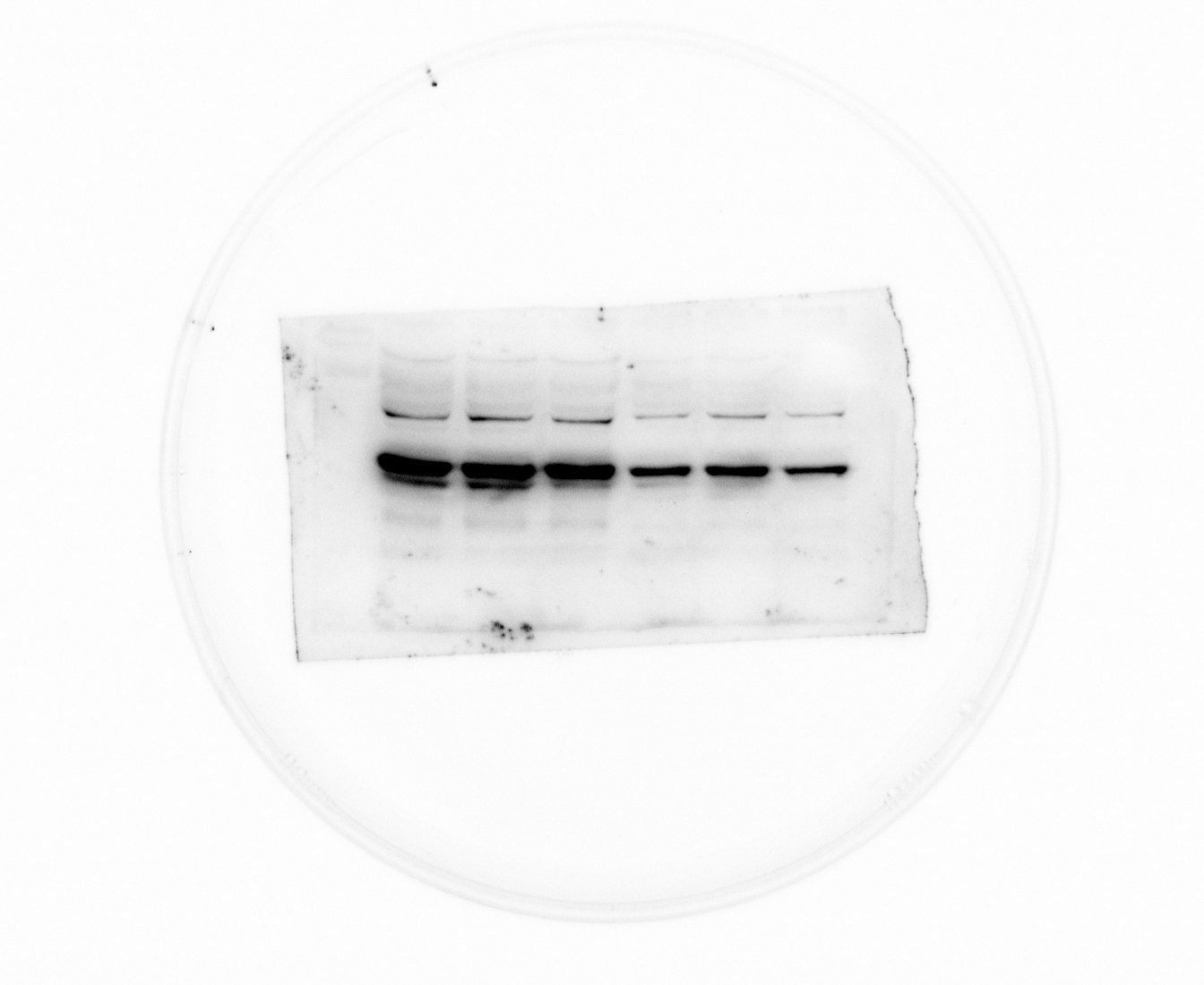
**

**8C- N-Cadherin**

**
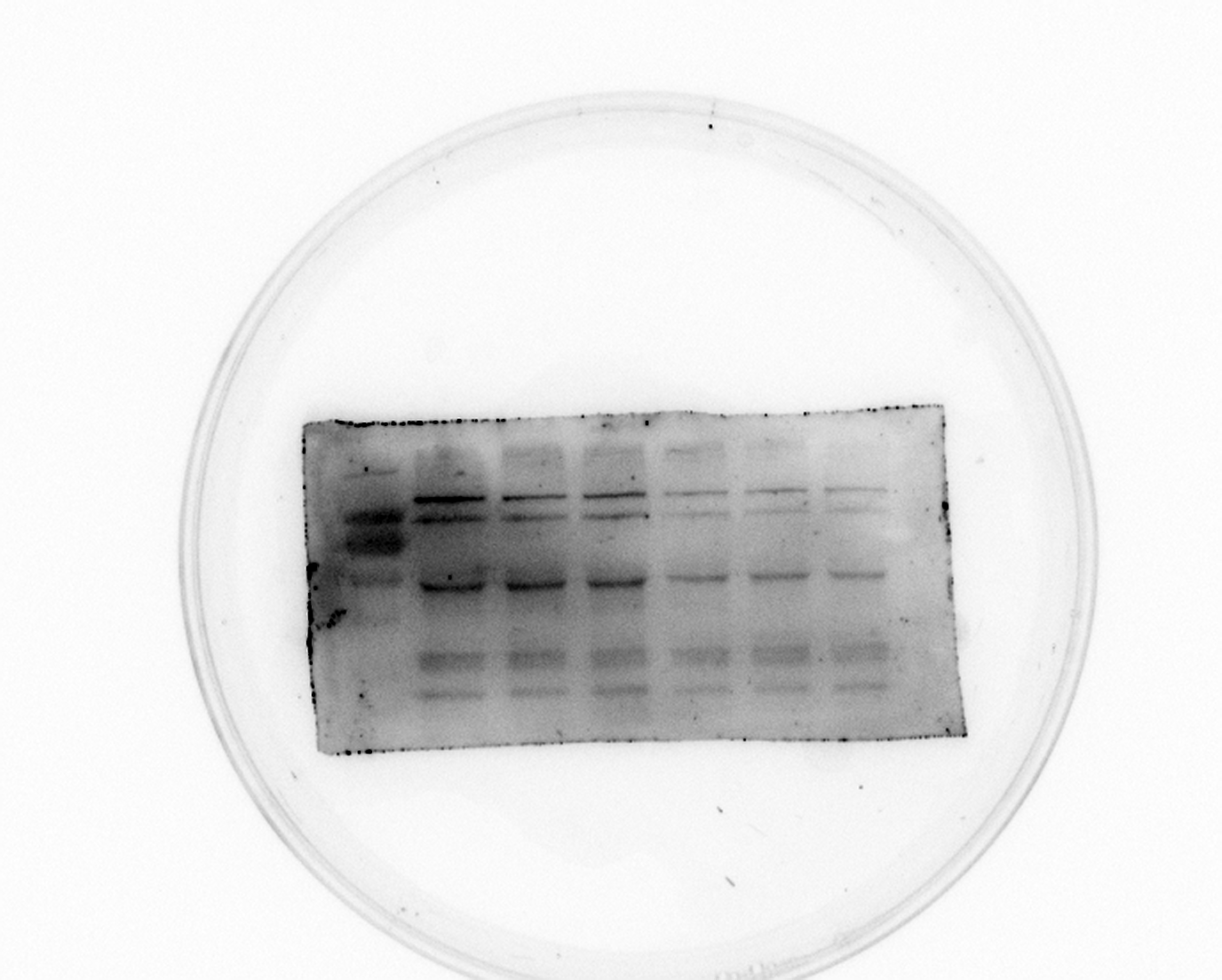
**

**8C-Vimentin**

**
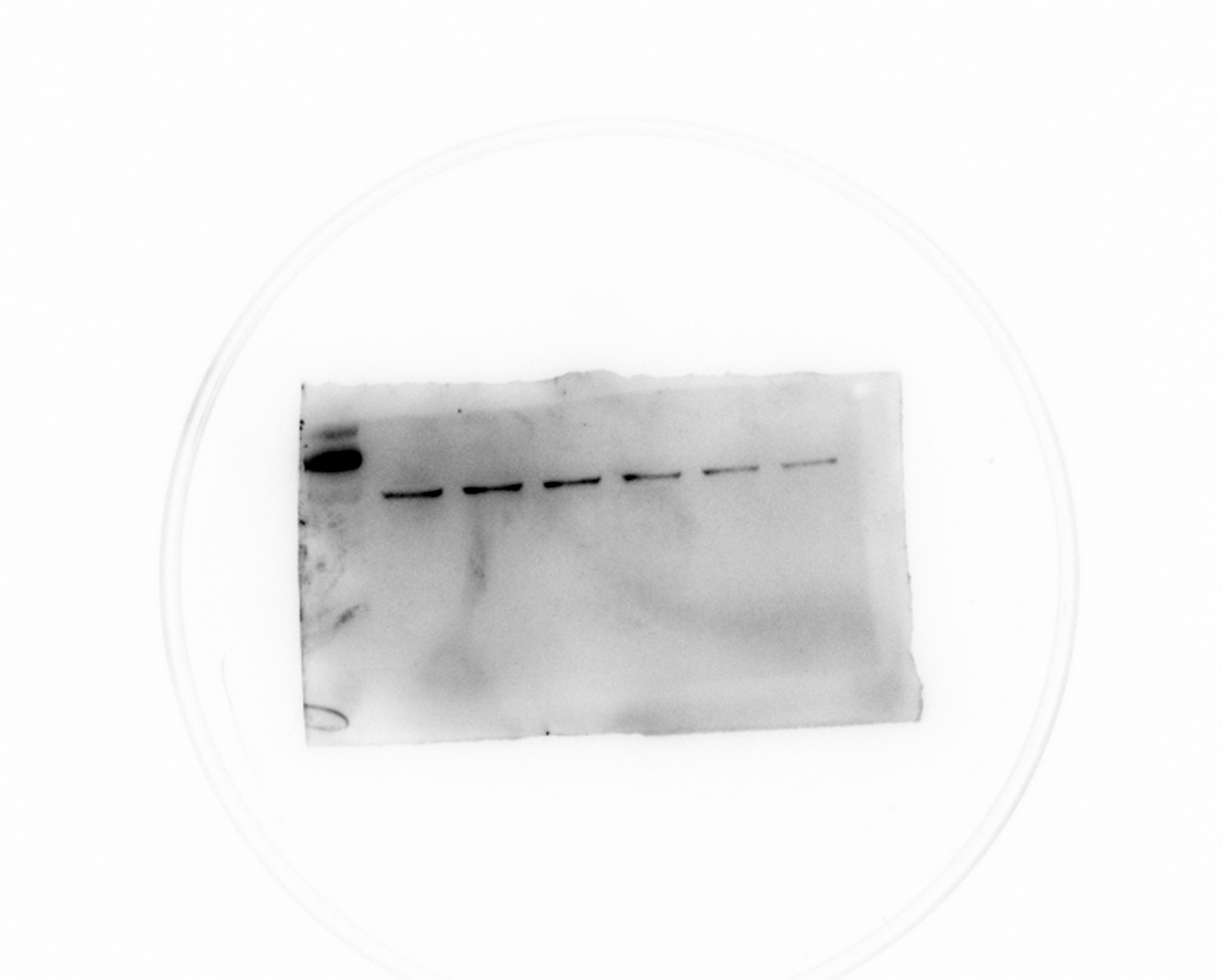
**
