# Supplementary material for: Expression and Gene Regulation Network of Adenosine Receptor A2B in Lung Adenocarcinoma: A Potential Diagnostic and Prognostic Biomarker
Source: Front Mol Biosci. 2021 Jul 19;8:663011. doi: 10.3389/fmolb.2021.663011 (PMC8326519; doi:10.3389/fmolb.2021.663011)
Supplement: Supplementary file 5 [file Table3.DOCX]

Supplementary Table 3. The top 50 negatively and positively correlated genes

The top 50 positively correlated genes

| N0. | Query | Statistic | P-value | FDR (BH) | Event_SD | Event_TD |
| --- | --- | --- | --- | --- | --- | --- |
| 1 | ADORA2B | 1 | 1.00E-33 | 1.00E-29 | 515 | 515 |
| 2 | ANXA1 | 0.493247748 | 6.25E-33 | 6.25E-29 | 515 | 515 |
| 3 | ITGA3 | 0.464200142 | 6.96E-29 | 4.64E-25 | 515 | 515 |
| 4 | S100A6 | 0.461050202 | 1.81E-28 | 9.06E-25 | 515 | 515 |
| 5 | RAB38 | 0.446652094 | 1.28E-26 | 5.10E-23 | 515 | 515 |
| 6 | TMEM43 | 0.441861525 | 5.03E-26 | 1.67E-22 | 515 | 515 |
| 7 | FAS | 0.429879907 | 1.41E-24 | 4.03E-21 | 515 | 515 |
| 8 | RAB27B | 0.422604007 | 1.00E-23 | 2.50E-20 | 515 | 515 |
| 9 | ARL6IP5 | 0.418938575 | 2.64E-23 | 5.87E-20 | 515 | 515 |
| 10 | SPRY2 | 0.418216482 | 3.20E-23 | 6.39E-20 | 515 | 515 |
| 11 | GNA15 | 0.415357962 | 6.74E-23 | 1.23E-19 | 515 | 515 |
| 12 | TXNDC17 | 0.413071863 | 1.22E-22 | 1.87E-19 | 515 | 515 |
| 13 | MPZL2 | 0.412242183 | 1.51E-22 | 2.01E-19 | 515 | 515 |
| 14 | MYOF | 0.41130626 | 1.92E-22 | 2.39E-19 | 515 | 515 |
| 15 | SGMS2 | 0.411083636 | 2.03E-22 | 2.39E-19 | 515 | 515 |
| 16 | MET | 0.410530059 | 2.34E-22 | 2.60E-19 | 515 | 515 |
| 17 | LGALS3 | 0.410224635 | 2.53E-22 | 2.66E-19 | 515 | 515 |
| 18 | ASPH | 0.409303963 | 3.20E-22 | 3.20E-19 | 515 | 515 |
| 19 | TIPARP | 0.408741775 | 3.69E-22 | 3.51E-19 | 515 | 515 |
| 20 | MDFIC | 0.403041005 | 1.55E-21 | 1.29E-18 | 515 | 515 |
| 21 | TMEM159 | 0.402702462 | 1.69E-21 | 1.35E-18 | 515 | 515 |
| 22 | GPR110 | 0.402397475 | 1.82E-21 | 1.40E-18 | 515 | 513 |
| 23 | CLDN12 | 0.401607277 | 2.21E-21 | 1.64E-18 | 515 | 515 |
| 24 | MMP28 | 0.40088524 | 2.65E-21 | 1.89E-18 | 515 | 514 |
| 25 | SH3RF2 | 0.396968411 | 6.94E-21 | 4.47E-18 | 515 | 515 |
| 26 | WNT7B | 0.394942781 | 1.14E-20 | 7.09E-18 | 515 | 515 |
| 27 | S100A10 | 0.394700794 | 1.20E-20 | 7.29E-18 | 515 | 515 |
| 28 | CAV2 | 0.39409615 | 1.39E-20 | 8.03E-18 | 515 | 515 |
| 29 | RBKS | 0.394061381 | 1.41E-20 | 8.03E-18 | 515 | 515 |
| 30 | SRD5A3 | 0.391782483 | 2.43E-20 | 1.28E-17 | 515 | 515 |
| 31 | MAPKAPK3 | 0.39168829 | 2.49E-20 | 1.28E-17 | 515 | 515 |
| 32 | REEP6 | 0.387807157 | 6.28E-20 | 2.92E-17 | 515 | 515 |
| 33 | IVL | 0.387471419 | 6.79E-20 | 3.09E-17 | 515 | 451 |
| 34 | STEAP3 | 0.386815406 | 7.93E-20 | 3.52E-17 | 515 | 515 |
| 35 | IL27RA | 0.386298526 | 8.96E-20 | 3.89E-17 | 515 | 515 |
| 36 | PON2 | 0.385394782 | 1.11E-19 | 4.61E-17 | 515 | 515 |
| 37 | PLS3 | 0.382533371 | 2.16E-19 | 8.47E-17 | 515 | 515 |
| 38 | WBSCR26 | 0.382021601 | 2.43E-19 | 9.36E-17 | 515 | 506 |
| 39 | SDC4 | 0.381826988 | 2.55E-19 | 9.61E-17 | 515 | 515 |
| 40 | CST6 | 0.381215693 | 2.93E-19 | 1.09E-16 | 515 | 514 |
| 41 | PHLDA3 | 0.379359349 | 4.50E-19 | 1.55E-16 | 515 | 515 |
| 42 | CAST | 0.378185202 | 5.90E-19 | 1.96E-16 | 515 | 515 |
| 43 | PITPNM3 | 0.377510345 | 6.88E-19 | 2.25E-16 | 515 | 515 |
| 44 | ACTBL2 | 0.377357256 | 7.12E-19 | 2.30E-16 | 515 | 462 |
| 45 | NT5E | 0.376437299 | 8.79E-19 | 2.79E-16 | 515 | 515 |
| 46 | HPGD | 0.376224449 | 9.22E-19 | 2.88E-16 | 515 | 515 |
| 47 | SPRED1 | 0.375891255 | 9.95E-19 | 3.06E-16 | 515 | 515 |
| 48 | DPY19L1 | 0.374646667 | 1.32E-18 | 3.99E-16 | 515 | 515 |
| 49 | CRTAP | 0.37402469 | 1.52E-18 | 4.53E-16 | 515 | 515 |
| 50 | AREG | 0.371664972 | 2.58E-18 | 7.58E-16 | 515 | 515 |

The top 50 negatively correlated genes

| N0. | Query | Statistic | P-value | FDR (BH) | Event_SD | Event_TD |
| --- | --- | --- | --- | --- | --- | --- |
| 1 | KLHL25 | -0.413106324 | 1.21E-22 | 1.87E-19 | 515 | 515 |
| 2 | MSI1 | -0.412656217 | 1.36E-22 | 1.94E-19 | 515 | 510 |
| 3 | KDM2B | -0.407771424 | 4.72E-22 | 4.29E-19 | 515 | 515 |
| 4 | NEB | -0.403607232 | 1.35E-21 | 1.17E-18 | 515 | 515 |
| 5 | CBFA2T2 | -0.399432534 | 3.79E-21 | 2.61E-18 | 515 | 515 |
| 6 | PPM1H | -0.39734031 | 6.33E-21 | 4.22E-18 | 515 | 515 |
| 7 | RET | -0.393585128 | 1.58E-20 | 8.76E-18 | 515 | 515 |
| 8 | CALCB | -0.391764953 | 2.44E-20 | 1.28E-17 | 515 | 263 |
| 9 | IDH2 | -0.39046767 | 3.33E-20 | 1.64E-17 | 515 | 515 |
| 10 | DNMT3A | -0.390437079 | 3.36E-20 | 1.64E-17 | 515 | 515 |
| 11 | KIF5C | -0.388640075 | 5.15E-20 | 2.45E-17 | 515 | 515 |
| 12 | C1orf115 | -0.385440911 | 1.10E-19 | 4.61E-17 | 515 | 515 |
| 13 | F7 | -0.382957734 | 1.96E-19 | 7.99E-17 | 515 | 477 |
| 14 | C1orf95 | -0.382758469 | 2.05E-19 | 8.20E-17 | 515 | 491 |
| 15 | C12orf34 | -0.380580783 | 3.40E-19 | 1.24E-16 | 515 | 515 |
| 16 | ENO3 | -0.380015027 | 3.87E-19 | 1.38E-16 | 515 | 515 |
| 17 | NEURL | -0.379521682 | 4.34E-19 | 1.52E-16 | 515 | 512 |
| 18 | CALCA | -0.37880485 | 5.11E-19 | 1.73E-16 | 515 | 420 |
| 19 | KIF1A | -0.370079313 | 3.68E-18 | 1.06E-15 | 515 | 488 |
| 20 | MARK1 | -0.369903504 | 3.82E-18 | 1.09E-15 | 515 | 515 |
| 21 | MTMR7 | -0.369517957 | 4.16E-18 | 1.16E-15 | 515 | 515 |
| 22 | ATOH7 | -0.36873982 | 4.95E-18 | 1.34E-15 | 515 | 422 |
| 23 | SEC61A2 | -0.367307058 | 6.79E-18 | 1.76E-15 | 515 | 515 |
| 24 | PPARGC1A | -0.366411859 | 8.27E-18 | 2.12E-15 | 515 | 514 |
| 25 | MLLT11 | -0.365873937 | 9.31E-18 | 2.32E-15 | 515 | 515 |
| 26 | ASCL1 | -0.364680863 | 1.21E-17 | 2.95E-15 | 515 | 466 |
| 27 | RNFT2 | -0.362450231 | 1.96E-17 | 4.56E-15 | 515 | 515 |
| 28 | SMARCD1 | -0.360970351 | 2.70E-17 | 6.14E-15 | 515 | 515 |
| 29 | LOC441177 | -0.358939439 | 4.18E-17 | 8.82E-15 | 515 | 177 |
| 30 | PHGDH | -0.355398444 | 8.89E-17 | 1.81E-14 | 515 | 515 |
| 31 | CPT1A | -0.353743567 | 1.26E-16 | 2.52E-14 | 515 | 515 |
| 32 | PLEKHG5 | -0.353228173 | 1.40E-16 | 2.78E-14 | 515 | 515 |
| 33 | GPT2 | -0.351091408 | 2.19E-16 | 4.22E-14 | 515 | 515 |
| 34 | PAH | -0.350673334 | 2.39E-16 | 4.56E-14 | 515 | 399 |
| 35 | SYCP2L | -0.350228288 | 2.62E-16 | 4.90E-14 | 515 | 478 |
| 36 | ATP6V1B1 | -0.349453083 | 3.08E-16 | 5.70E-14 | 515 | 515 |
| 37 | SLC38A8 | -0.347070054 | 5.04E-16 | 9.07E-14 | 515 | 232 |
| 38 | CCNJ | -0.345909046 | 6.39E-16 | 1.13E-13 | 515 | 515 |
| 39 | GNAZ | -0.343991608 | 9.44E-16 | 1.63E-13 | 515 | 515 |
| 40 | PHF8 | -0.343790029 | 9.84E-16 | 1.68E-13 | 515 | 515 |
| 41 | SMAP1 | -0.34288717 | 1.18E-15 | 1.98E-13 | 515 | 515 |
| 42 | GFOD2 | -0.342236375 | 1.35E-15 | 2.22E-13 | 515 | 515 |
| 43 | KIAA0182 | -0.341879667 | 1.45E-15 | 2.32E-13 | 515 | 515 |
| 44 | KIAA1549 | -0.34141032 | 1.59E-15 | 2.50E-13 | 515 | 515 |
| 45 | NTNG2 | -0.340272577 | 2.00E-15 | 3.05E-13 | 515 | 513 |
| 46 | RNF183 | -0.340101616 | 2.07E-15 | 3.13E-13 | 515 | 499 |
| 47 | MTERFD3 | -0.339225621 | 2.46E-15 | 3.67E-13 | 515 | 515 |
| 48 | C13orf23 | -0.337635603 | 3.38E-15 | 4.83E-13 | 515 | 515 |
| 49 | NFYB | -0.337326859 | 3.59E-15 | 5.09E-13 | 515 | 515 |
| 50 | CCAR1 | -0.336600972 | 4.15E-15 | 5.76E-13 | 515 | 515 |
