# Supplementary material for: Expression and Gene Regulation Network of Adenosine Receptor A2B in Lung Adenocarcinoma: A Potential Diagnostic and Prognostic Biomarker
Source: Front Mol Biosci. 2021 Jul 19;8:663011. doi: 10.3389/fmolb.2021.663011 (PMC8326519; doi:10.3389/fmolb.2021.663011)
Supplement: Supplementary file 7 [file Table4.DOCX]

**Data Availability Statements**

### Okayama Lung data was downloaded from the Oncomine database under the Series accession number [GSE31210](https://www.ncbi.nlm.nih.gov/geo/query/acc.cgi?acc=GSE31210) (<http://www.ncbi.nlm.nih.gov/geo/query/acc.cgi?acc=GSE31210>). Landi Lung data was downloaded from the Oncomine database under the Series accession number [GSE10072](https://www.ncbi.nlm.nih.gov/geo/query/acc.cgi?acc=GSE10072) (<http://www.ncbi.nlm.nih.gov/geo/query/acc.cgi?acc=GSE10072>). Selamat Lung data was downloaded from the Oncomine database under the Series accession number [GSE32863](https://www.ncbi.nlm.nih.gov/geo/query/acc.cgi?acc=GSE32863) (<http://www.ncbi.nlm.nih.gov/geo/query/acc.cgi?acc=GSE32863>).

### Stearman Lung data was downloaded from the Oncomine database under the Series accession number [GSE2514](https://www.ncbi.nlm.nih.gov/geo/query/acc.cgi?acc=GSE2514) (<http://www.ncbi.nlm.nih.gov/geo/query/acc.cgi?acc=GSE2514>). Beer Lung data was downloaded from the Oncomine database under the Array Type: HumanGeneFL Array, Measured 5,338 genes, 7,133 reporters (Beer, D. G., Kardia, S. L., Huang, C. C., Giordano, T. J., Levin, A. M., Misek, D. E., et al.(2002). Gene-expression profiles predict survival of patients with lung adenocarcinoma. Nat. Med. 8, 816-824. doi: 10.1038/nm733). Su Lung data was downloaded from the Oncomine database under the Series accession number [GSE7670](https://www.ncbi.nlm.nih.gov/geo/query/acc.cgi?acc=GSE7670) (<http://www.ncbi.nlm.nih.gov/geo/query/acc.cgi?acc=GSE7670>). Bhattacharjee Lung was downloaded from the Oncomine database under the Array Type: Human Genome U95A-Av2 Array, Measured 8,603 genes, 12,651 reporters (Bhattacharjee, A., Richards, W. G., Staunton, J., Li, C., Monti, S., Vasa, P., et al. (2001). Classification of human lung carcinomas by mRNA expression profiling reveals distinct adenocarcinoma subclasses. Proc. Natl. Acad. Sci. U S A98, 13790-13795. doi: 10.1073/pnas.191502998). Garber Lung datasets data was downloaded from the Oncomine database under the Series accession number [GSE3398](http://www.ncbi.nlm.nih.gov/geo/query/acc.cgi?acc=GSE3398) (<http://www.ncbi.nlm.nih.gov/geo/query/acc.cgi?acc=GSE3398>). OncoPrint data was downloaded from the cBioPortal database Shortened URL [https://bit.ly/3oTsOd5.](https://bit.ly/2N2NJNG.) Mutation data was downloaded from the cBioPortal database Shortened URL <https://bit.ly/2ObTF7t.> Clinical attribute data was downloaded from the cBioPortal database Shortened URL <https://bit.ly/3rnelHU.> LinkedInkOmics data was downloaded from the LinkedInkOmics database under the ID number ID-62189.
